# Off-tumor IDO1 target engagements determine the cancer-immune set point and predict the immunotherapeutic efficacy

**DOI:** 10.1136/jitc-2021-002616

**Published:** 2021-06-20

**Authors:** Lin Xie, Kuan Hu, Yanhong Duo, Takashi Shimokawa, Katsushi Kumata, Yiding Zhang, Cuiping Jiang, Lulu Zhang, Nobuki Nengaki, Hidekatsu Wakizaka, Yihai Cao, Ming-Rong Zhang

**Affiliations:** 1 Department of Advanced Nuclear Medicine Sciences, National Institute of Radiological Sciences, National Institutes for Quantum and Radiological Science and Technology, Chiba, Japan; 2 Department of Microbiology, Tumor and Cell Biology, Karolinska Institute, Stockholm, Sweden; 3 Department of Accelerator and Medical Physics, National Institute of Radiological Sciences, National Institutes for Quantum and Radiological Science and Technology, Chiba, Japan

**Keywords:** tumor biomarkers, immunomodulation, biomarkers, tumor, combined modality therapy, indoleamine-pyrrole 2, 3-dioxygenase

## Abstract

**Background:**

Indoleamine-2,3-dioxygenase 1 (IDO1) has been intensively pursued as a therapeutic target to reverse the immunosuppressive cancer-immune milieu and promote tumor elimination. However, recent failures of phase III clinical trials with IDO1 inhibitors involved in cancer immunotherapies highlight the urgent need to develop appropriate methods for tracking IDO1 when the cancer-immune milieu is therapeutically modified.

**Methods:**

We utilized a small-molecule radiotracer, ^11^C-l-1MTrp, to quantitatively and longitudinally visualize whole-body IDO1 dynamics. Specifically, we first assessed ^11^C-l-1MTrp in mice-bearing contralateral human tumors with distinct IDO1 expression patterns. Then, we applied ^11^C-l-1MTrp to longitudinally monitor whole-body IDO1 variations in immunocompetent melanoma-bearing mice treated with 1-methyl-l-tryptophan plus either chemotherapeutic drugs or antibodies targeting programmedcell death 1 and cytotoxic T-lymphocyte-associated protein 4.

**Results:**

^11^C-l-1MTrp positron emission tomography (PET) imaging accurately delineated IDO1 expression in xenograft mouse models. Moreover, we were able to visualize dynamic IDO1 regulation in the mesenteric lymph nodes (MLNs), an off-tumor IDO1 target, where the percentage uptake of ^11^C-l-1MTrp accurately annotated the therapeutic efficacy of multiple combination immunotherapies in preclinical models. Remarkably, ^11^C-l-1MTrp signal intensity in the MLNs was inversely related to the specific growth rates of treated tumors, suggesting that IDO1 expression in the MLNs can serve as a new biomarker of the cancer-immune set point.

**Conclusions:**

PET imaging of IDO1 with ^11^C-l-1MTrp is a robust method to assess the therapeutic efficacy of multiple combinatorial immunotherapies, improving our understanding of the merit and challenges of IDO1 regimens. Further validation of this animal data in humans is ongoing. We envision that our results will provide a potential precision medicine paradigm for noninvasive visualizing each patient’s individual response in combinatorial cancer immunotherapy, and tailoring optimal personalized combination strategies.

## Introduction

Indoleamine-2,3-dioxygenase 1 (IDO1), a rate-limiting tryptophan (Trp)-degrading enzyme, has been intensively pursued as a drug target to reverse the immunosuppressive cancer-immune milieu and promote tumor elimination.^1 2^ As an important immunoregulator, IDO1 not only triggers immunosuppressive mechanisms but also orchestrates the immune equilibrium of individuals.^3 4^ Resembling programmed death-ligand 1 (PD-L1), the expression and activity of IDO1 in the immune compartment is tightly regulated. It is highly expressed in inflamed tissues, with its expression induced (mainly in myeloid cells) by the proinflammatory cytokines such as interferon-gamma (IFN-γ) and interleukin-6, to control excessive immune reactions via local Trp depletion and production of proapoptotic Trp catabolites. IDO1 is also broadly expressed in a wide spectrum of human tumors, including melanoma, gynecological cancers, colon cancer, and hematological malignancies, and its expression is associated with poor clinical outcome.^5^

Various IDO1 inhibitors have been developed and evaluated in clinical trials, either as a monotherapy or in combination with chemotherapy, radiotherapy, or immune checkpoint blockade.^6 7^ It had been widely anticipated that the leading IDO1 inhibitor, such as INCB024360 (Epacadostat),^8^ would gain regulatory approval because of the very promising outcomes seen in preclinical and early-phase clinical studies.^9 10^ However, one phase III trial in patients with unresectable or metastatic melanoma delivered unsatisfactory results (the ECHO-301 trial; NCT02752074),^11^ which instigated the termination of at least nine clinical trials evaluating IDO1-based regimens in multiple cancers.^3 11^ Although probable reasons for such failures include patient population selection criteria and dosing and therapeutic regimens, the validity of IDO1 as a target also needs to be considered.

Accurate assessment of IDO1 alteration and activity is crucial for determining the pharmaco-biological activity of IDO1 therapeutic strategies. Analysis of kynurenine and Trp levels is commonly used to measure in vivo IDO1 activity during treatment with IDO1-blockade combinatorial immunotherapies.^12^ However, a change in the Trp catabolism level provides limited useful information because this parameter represents circulating Trp metabolism, which can be confounded by exogenous influences such as dietary intake. In addition, although direct quantification of IDO1 in tumor biopsy samples has also been performed, post-translational modifications of the IDO1 enzyme alter its activity,^13^ and the uncertainties caused by sampling variations and the heterogeneous expression of IDO1 cause immunohistochemical or messenger RNA (mRNA) profiling of tumor tissue to be unreliable. The fact that IDO1 is highly inducible and alterable highlights the need for new methods that can noninvasively and quantitatively track IDO1 in living bodies subjected to immunotherapeutic interventions. Visualization of drug engagement and its correlation with immunoediting efficacy would provide direct evidence for improving the therapeutic outcomes of patients with cancer receiving IDO1-blockade in a therapeutic setting.

Functional positron emission tomography (PET) imaging is a potentially ideal tool for capturing real-time variations in IDO1 content, because it enables noninvasive, highly sensitive, repetitive, and quantitative imaging of positron-emitting, target-specific probes.^14^ The utilization of PET imaging to study the relationship between IDO1 expression and immunotherapy efficacy has never been reported, despite several IDO1 inhibitors having been radiolabeled for use in pharmacokinetic studies^15 16^ and to perform IDO1-targeted cancer imaging.^17 18^

Considering the critical roles of IDO1 in cancer metabolism and immune modulation,^19^ we envisioned that IDO1 holds potential as an effective biomarker to identify the cancer-immune set point, which might help to specify the right patient at the right time for IDO1-blockade combination therapy. Here, to test this hypothesis, we utilized an IDO1-specific PET tracer to define the relationship between IDO1 expression and the therapeutic response to IDO1 blockade. Specifically, we highlight the development cycle starting from tracer synthesis (^11^C-l-1MTrp) and biomarker identification (IDO1 in the mesenteric lymph nodes (MLNs)) to biomarker validation in a mouse model of melanoma treated with different immunotherapy regimens. Moreover, we demonstrate the potential clinical utility of this off-tumor imaging platform by illustrating its predictive value for the cancer-immune set point across multiple combinatorial immunotherapeutic strategies.

## Methods

### Radiosynthesis of ^11^C-l-1MTrp

The radioisotope ^11^C was produced by a ^14^N (p, α) ^11^C nuclear reaction using a CYPRIS HM-18 cyclotron (Sumitomo Heavy Industry, Tokyo, Japan). Introducing ^11^C into the indole ring of 1-Methyl-l-tryptophan (l-1MTrp) produced ^11^C-l-1MTrp as injectable solutions in 40±2 min according to our previously reported method.^16^ These radiotracers had a molar activity of 47–130 GBq/μmol and >98% radiochemical purity (n=80) and were provided for all experiments.

### Tumor models

Human tumor xenograft model: to facilitate comparison of the IDO1 levels and ^11^C-l-1MTrp uptake, two human cell lines, the NCI-H69 small cell lung cancer and MDA-MB231 breast cancer, were selected for PET/CT imaging and biodistribution studies. Tumor xenograft models were established via subcutaneous injection of NCI-H69 (2.5×10^6^) and MDA-MB231 (2.5×10^6^) cells into the left and right flanks, respectively, of immunodeficient BALB/c nude mice (Japan SLC, Shizuoka, Japan) in a total volume of 0.1 mL serum-free medium containing 50% Matrigel (BD Biosciences, San Jose, California, USA). The cells were analyzed by quantitative real-time reverse transcriptase-polymerase chain reaction (qRT-PCR) before injection to confirm their *IDO1* expression patterns. When the tumors grew to a maximum tumor diameter of 9–12 mm, the mice were selected randomly for further studies.

### PET/CT scans

Whole-body dynamic PET scans were performed after intravenous injection of ^11^C-l-1MTrp (16.2–19.5 MBq/0.1 mL, corresponding to 0.73–1.45 nmol of l-1MTrp) using a small-animal Siemens Inveon PET scanner (Siemens, Knoxville, Tennessee, USA) to acquire 159 transaxial slices with 0.796 mm (center-to-center) spacing, a 10 cm transaxial field of view (FOV), and a 12.7 cm axial FOV. Emission scans of the tumor models were acquired in three-dimensional list mode with an energy window of 350–650 keV under isoflurane anesthesia from 0 to 75 min after the injection.

### Ex vivo biodistribution studies

After ^11^C-l-1MTrp (7.1–7.5 MBq/0.1 mL, corresponding to 0.44–0.88 nmol of l-1MTrp) intravenous injection, human xenograft tumor-bearing mice were sacrificed by cervical dislocation at 5, 15, 30, 60, and 90 min, while combinatorial immunotherapy-treated syngeneic mice were sacrificed by cervical dislocation at 60 min. Tumors and major tissues (blood, tumor, MLN, heart, lung, thymus, liver, pancreas, spleen, kidney, adrenal, intestine, muscle, testis, epididymis, and brain) were promptly excised, harvested, and weighed. Radioactivity was counted using a γ-counter, and results are expressed as %ID/g tissue. All radioactivity measurements were corrected for decay.

## Results

### Radiotracer ^11^C-l-1MTrp specifically accumulates in IDO1-expressing human tumors

**Figure 1:**
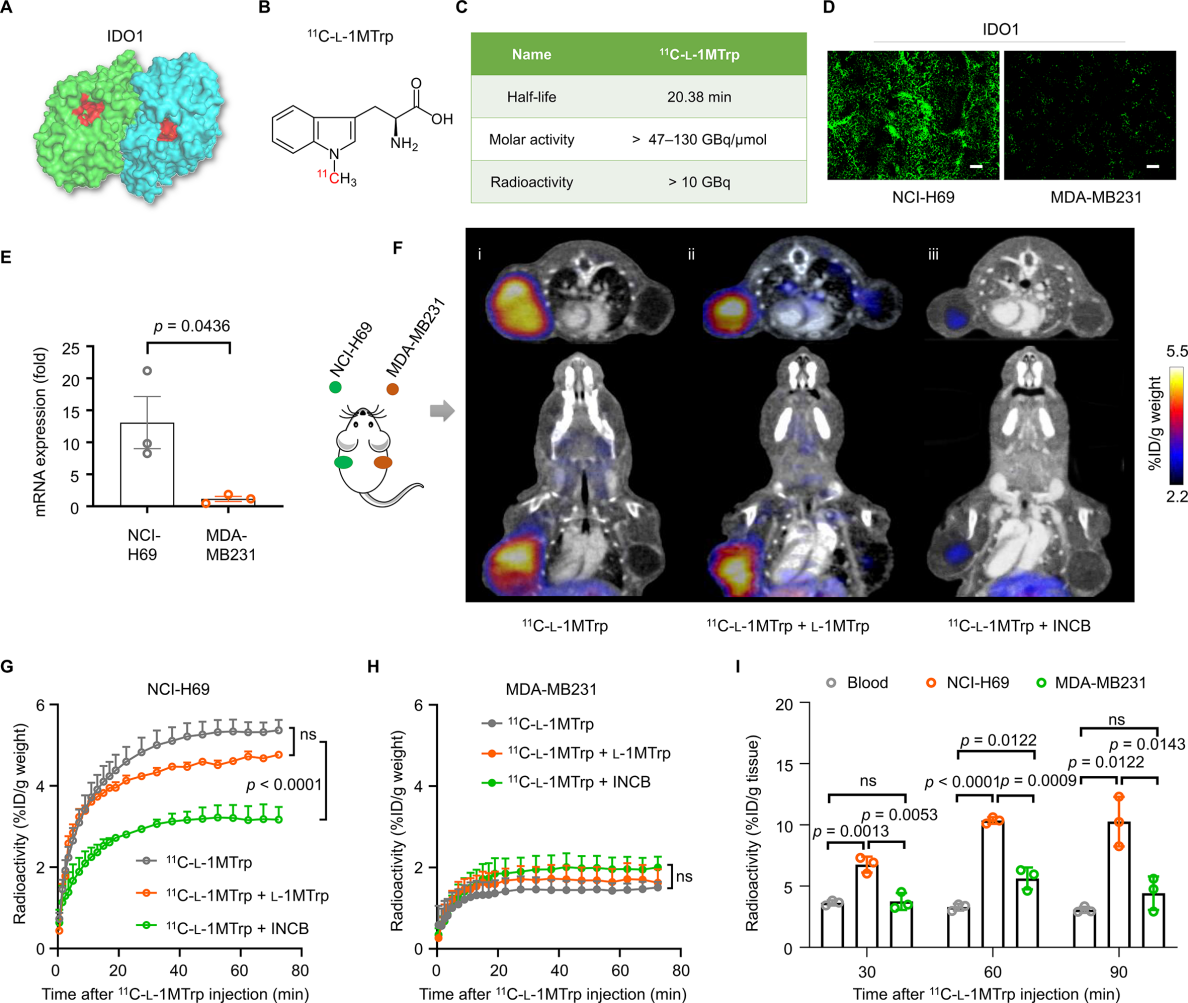
^11^C-l-1MTrp imaging of IDO1 in human tumor models. (A) Protein structure of IDO1 (PDB ID: 2D0U). The blue and green colors indicate the subunits of the IDO1 dimer, and the red color indicates the tryptophan catalytic site. (B) Chemical structure of ^11^C-l-1MTrp. (C) Radio-characteristics of ^11^C-l-1MTrp. The half-life indicates the physical half-life of ^11^C-l-1MTrp. The molar activity and radioactivity were determined at the end of the synthesis (n=80) and showed an averaged synthesis time of 40 min from the end of the bombardment. (D) Immunostaining for IDO1 expression in tumor tissue samples from mice subcutaneously injected with 2.5×10^6^ NCI-H69 or MDA-MB231 cells. Green: Alexa Fluor 488-labeled anti-IDO1 antibody. Scale bar: 100 µm. (E) Quantification of IDO1 expression in NCI-H69 and MDA-MB-231 cells based on quantitative real-time reverse transcriptase-PCR. (F) Representative PET/CT images of tumor-bearing mice. Tumor positions: NCI-H69 tumor in the left flank and MDA-MB231 tumor in the right flank. Signal lower than 2.2% ID/g weight was subtracted of all PET images. (i) Representative coregistered ^11^C-l-1MTrp PET/CT image; (ii) Competition ^11^C-l-1MTrp PET/CT images after a coinjection of the ‘cold (opposite to the radiolabeled ^11^C-l-1MTrp)’ IDO1 inhibitor l-1MTrp (50 mg/kg) or (iii) INCB (INCB024360, 10 mg/kg). PET images were summed from 60 min to 75 min after ^11^C-l-1MTrp injection. (G, H) Time–activity curves of ^11^C-l-1MTrp in (G) NCI-H69 and (H) MDA-MB231 xenografts. (I) Ex vivo biodistribution data collected at 30, 60, and 90 min after ^11^C-l-1MTrp injection into xenograft mice. All comparisons were performed using an unpaired two-tailed Student’s t-test. Data represent the mean±SEM, n=3. IDO1, indoleamine-2,3-dioxygenase 1; PET, positron emission tomography.

l-1MTrp is a well-known IDO1 inhibitor showing specific binding to the catalytic pocket of IDO1, and has been indicated to inhibit tumor growth in diverse tumor types in vivo (figure 1A).^20^ Radiotracer ^11^C-l-1MTrp was synthesized with satisfactory radio-characteristics (figure 1B and C).^16^ To study the potential of ^11^C-l-1MTrp to specifically visualize IDO1 in human tumors, we performed ^11^C-l-1MTrp PET imaging in mice inoculated with human tumors with different IDO1 expression levels. Immunohistological staining for IDO1 in tumor sections indicated that the NCI-H69 small cell lung cancer and MDA-MB231 breast cancer markedly differed in their IDO1 expression levels, with higher IDO1 expression observed in the NCI-H69 than in the MDA-MB231 (figure 1D). This trend was also observed at the mRNA level by qRT-PCR (figure 1E).

PET/CT imaging showed strong uptake of ^11^C-l-1MTrp in the NCI-H69 xenografts but negligible tracer uptake in the MDA-MB231 xenografts (figure 1F–I), in good accordance with their IDO1 expression patterns. We also quantified the radioactivity levels in the tumor site based on the dynamic PET and corresponding CT images at 0–75 min after injection. The tracer uptake in NCI-H69 showed as 5.36%±0.51 % ID/g weight (the percentage value is quantified for *t*=75 min after injection, similarly hereinafter) when the radioactivity in tumor is steady (figure 1G), significantly higher than that in MDA-MB231 (1.51%±0.23% ID/g weight) (figure 1H).

To verify the specific binding of ^11^C-l-1MTrp to IDO1, we then treated the same mouse model using two well-known IDO1 inhibitors, l-1MTrp (50 mg/kg) and INCB024360 (10 mg/kg),^8^ and acquired PET images after blockade by coinjection with the radiotracer. As expected, the uptake of ^11^C-l-1MTrp was significantly decreased in the NCI-H69 xenografts (3.17%±0.54% ID/g weight) of INCB024360-pretreated mice (figure 1Fiii, G). Surprisingly, l-1MTrp pretreatment had little effect on the uptake of the tracer in NCI-H69 xenografts (4.77%±0.09% ID/g weight, figure 1Fii, G). As l-1MTrp is an analog of the essential amino acid Trp,^3^ this suggests that l-1MTrp administration did not interfere with ^11^C-l-1MTrp PET while visualizing IDO1 in vivo.

An ex vivo biodistribution study in this xenograft model validated the PET results (figure 1I and online supplemental table 1). Peak uptake in NCI-H69 tumors was achieved at 60 min, and the tumor-to-blood ratio was estimated to be 2.5–3 (online supplemental figure 1A, B). These results demonstrated that ^11^C-l-1MTrp could specifically bind to human tumor-derived IDO1 and that its accumulation mimicked the trends in IDO1 expression observed in vivo.

10.1136/jitc-2021-002616.supp1 Supplementary data

### IDO1 blockade-containing combinatorial immunotherapy activates the antitumor immune response

**Figure 2:**
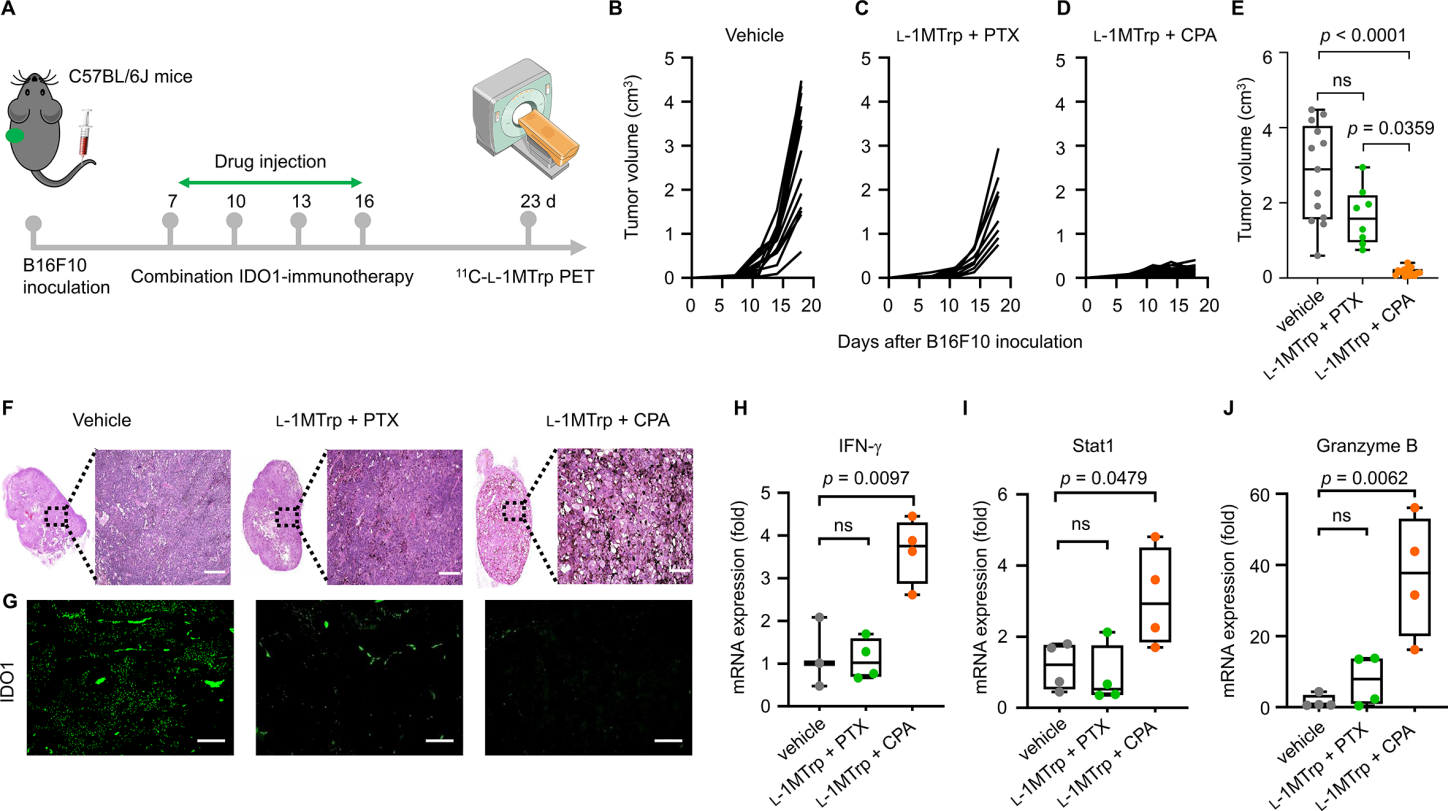
Antitumor effects of combination IDO1 blockade-containing regimens. (A) Schematic of the treatment plan. B16F10 tumor cells (5×10^4^) were inoculated into the left flank of C57BL/6J mice on day 0, and the mice were treated with vehicle, l-1MTrp (orally, 5 g/kg feed) + CPA (150 mg/kg) or l-1MTrp+PTX (13.3 mg/kg) on days 7, 10, 13, and 16 postinoculation. ^11^C-l-1MTrp PET/CT was performed on day 23. (B–D) Individual tumor growth curves of mice treated with vehicle, l-1MTrp+PTX, or l-1MTrp+CPA. (E) Tumor volume of mice on day 18 postinoculation. Data represent the mean±SEM, n=13 for vehicle group, n=8 for l-1MTrp+PTX group, and n=16 for l-1MTrp+CPA group. (F) H&E staining of tumor sections from treated mice on day 23 postinoculation. (G) Histochemical staining for the IDO1 protein in tumor sections from treated mice on day 23 postinoculation. Green indicates staining with an Alexa fluor 488-labeled anti-IDO1 antibody. (H–J) Quantification of the mRNA expression levels of several selected genes including interferon-gamma (IFN-γ), STAT1, and granzyme B in tumor tissues derived from the treated mice on day 23 postinoculaton. Data represent the mean±SEM, n=4. All comparisons were performed using an unpaired two-tailed Student’s t-test. CPA, cyclophosphamide; PTX, paclitaxel.

An IDO1-specific radiotracer that noninvasively and quantitatively tracks dynamic IDO1 expression during IDO1-blockade therapy would be expected to identify any divergence in individual responses to IDO1-targeted treatments. To test this hypothesis, we initially used ^11^C-l-1MTrp to evaluate therapeutic efficacy in B16F10 tumor-bearing immunocompetent mice, a commonly used preclinical tumor model with successful immunotherapies, receiving one of two clinically relevant IDO1 blockade-containing combinatorial therapy regimens, l-1MTrp (5 g/kg) plus cyclophosphamide (CPA, 150 mg/kg, an immunomodulatory chemical toxin^21^ or l-1MTrp plus paclitaxel (PTX, 13.3 mg/kg) (figure 2A). The results showed that at the end of therapy, the combination of CPA (intravenously, 150 mg/kg) with l-1MTrp (orally, 5 mg/kg feed) achieved >90% tumor growth inhibition in all mice. In contrast, PTX (intravenously, 13.3 mg/kg) plus l-1MTrp (orally, 5 mg/kg feed) showed only moderate antitumor efficacy (~50%) compared with vehicle or their respective monotherapies (figure 2B–E and online supplemental figure 2A–D), indicating the l-1MTrp+CPA combinatorial strategy induced a more effective antitumor response than that of l-1MTrp+PTX in the difficult-to-treat melanoma. Hematoxylin and eosin stain (H&E) staining of tumor sections revealed a large zone of cell necrosis in tissue from the l-1MTrp+CPA-treated mice, but this phenomenon was rarely observed in tumor samples from the l-1MTrp+PTX and vehicle-treated mice (figure 2F). We then stained tumor sections for the IDO1 protein and found that the highest IDO1 protein expression was detected in the vehicle group, while the lowest IDO1 protein level was in the l-1MTrp+CPA group (figure 2G and online supplemental figure 3A), indicating an obvious decrease in tumor IDO1 expression after effective IDO1 blockade-containing combinatorial therapy.

To elucidate whether the antitumor effect is caused by the re-establishment of anticancer immunity, we then analyzed the mRNA expression levels of several immune-related cytokines and proteins in tumors. *IFN-γ* expression was detected to be approximately fourfold higher in l-1MTrp+CPA-treated mice than in vehicle-treated and l-1MTrp+PTX-treated mice (figure 2H), indicating the activation of cytotoxic T cell-mediated and natural killer cell-mediated antitumor immunity.^22^ The mRNA expression of the immune mediator *stat1* (figure 2I) and effector molecule *granzyme B* (figure 2J), both of which are known to be responsive to IFN-γ treatment,^23 24^ was increased in the l-1MTrp+CPA-treated mice, leading to B16F10 tumor cell death. For mice treated with l-1MTrp+PTX, the mRNA expression of these genes was comparable to that in vehicle-treated mice. Collectively, our results are consistent with the idea that the l-1MTrp+CPA regimen is a robust therapeutic strategy for cancer immunotherapy, in which l-1MTrp blocks the tumor-derived IDO1 protein. CPA can augment the immune response by regulating cytotoxic T cells and natural killer cells,^20^ generating a synergistic effect between CPA and l-1MTrp, which contributes to reversing the immunosuppressive cancer-immune state and produces superior antitumor outcomes (online supplemental figure 3B).^25^

### ^11^C-l-1MTrp specifically binds to IDO1 in the MLNs

**Figure 3:**
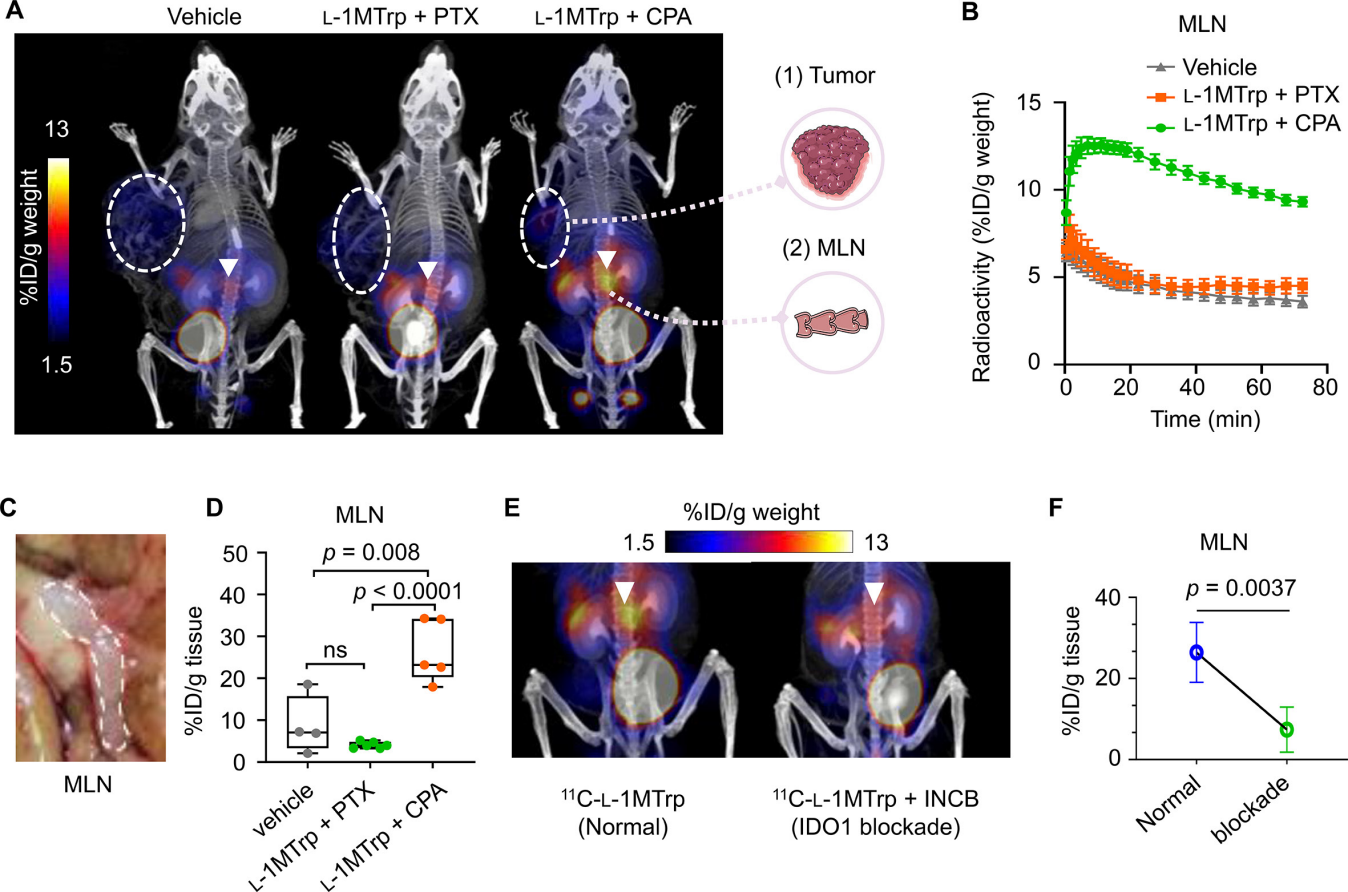
Whole-Body ^11^C-l-1MTrp PET/CT imaging of mice treated with IDO1 blockade-containing combinatorial therapy. B16F10 tumor cells (5×10^4^) were inoculated into the left flank of C57BL/6J mice at on day 0, and the mice were treated with vehicle, l-1MTrp (orally, 5 g/kg feed) + CPA (150 mg/kg) or l-1MTrp (orally, 5 g/kg feed) + PTX (13.3 mg/kg) on days 7, 10, 13, and 16 postinoculation. (A) representative PET/CT images of melanoma-bearing mice after treatment with vehicle, l-1MTrp+PTX, or l-1MTrp+CPA. Imaging was performed on day 23 after B16F10 cell inoculation, and ^11^C-l-1MTrp was injected intravenously. PET images were summed from 60 to 75 min postinjection. White circles indicate B16F10 tumors, and white triangles indicate the MLNs. Each PET/CT image is representative of at least three independent experiments. (B) Time–activity curves showing the dynamics of ^11^C-l-1MTrp in the MLNs of three groups of mice. (C) A photograph of the MLNs in a treated tumor-bearing mouse. (D) Ex vivo measurement of ^11^C-l-1MTrp in the MLNs of treated tumor-bearing mice at 60 min postinjection. Data represent the mean±SEM, n=4‒6 mice. (E) PET images after simultaneously blocking ^11^C-l-1MTrp accumulation in the MLNs with the IDO1-specific inhibitor INCB (INCB024360, 10 mg/kg). PET images were summed from 60 to 75 min after ^11^C-l-1MTrp injection. (F) Quantitative measurement of uptake (%ID/g tissue) in the MLNs by ex vivo biodistribution analysis after blocking with INCB024360. Data represent the mean±SEM, n=5 for the normal group and n=4 for the blockade group. All comparisons were performed using an unpaired two-tailed Student’s t-test. CPA, cyclophosphamide; MLN, mesenteric lymph node; PET, positron emission tomography; PTX, paclitaxel.

To examine whether ^11^C-l-1MTrp PET imaging can reflect the different treatment outcomes among the three treatment strategies (vehicle, l-1MTrp+PTX, and l-1MTrp+CPA), we performed whole-body dynamic PET imaging in B16F10-bearing mice treated with the three strategies. Figure 3A shows representative coregistered ^11^C-l-1MTrp PET/CT images, and very low background radioactivity was found in the brain, heart, lungs, liver, spleen, and intestine (online supplemental figure 4A, B); the radioactivity was rapidly eliminated via the bladder (online supplemental figure 4C), indicating a favorable pharmacokinetic profile that would be practical for daily practice. We detected no significant difference in ^11^C-l-1MTrp uptake by tumors among the three groups (figure 3Afigure 3Aand online supplemental figure 4D), although the l-1MTrp+CPA regimen exhibited a better antitumor efficacy than the other regimens, suggesting that direct imaging of IDO1 in tumors via ^11^C-l-1MTrp is not a suitable approach for predicting therapeutic outcomes. Surprisingly, in the mice treated with l-1MTrp+CPA, which showed the best antitumor efficacy (figure 2D), we observed extremely high tracer uptake in the MLNs (figure 3A, B and online supplemental video 1), an organ that has never been evaluated by PET imaging in cancer immunotherapy (figure 3C). In stark contrast, in vehicle-treated, single agent-treated and l-1MTrp+PTX-treated mice, only low background signals were found in the MLNs (figure 3A, B and online supplemental figure 2E, F). We conducted further ex vivo biodistribution assays to anatomically confirm the whole-body PET results. The radioactivity in the main organs was measured at 60 min postinjection of ^11^C-l-1MTrp and was consistent with the PET images (online supplemental table 2); the biodistribution results unambiguously demonstrated the highest uptake of ^11^C-l-1MTrp in the MLNs of the mice treated with l-1MTrp+CPA (23.93%±4.03% ID/g tissue), followed by the MLNs in the vehicle-treated mice (7.52%±3.86% ID/g tissue), and the lowest tracer uptake in the l-1MTrp+PTX-treated mice (4.28%±0.42% ID/g tissue) (figure 3D, online supplemental figure 2G and online supplemental table 2).

10.1136/jitc-2021-002616.supp2 Supplementary data

IDO1 plays crucial roles in balancing intestinal immune tolerance and gut microbiota maintenance, and IDO1 expression in the MLNs is inducible and dynamically changed to cope with challenges with foreign antigens derived from gut nutrients or the microbiota.^26^ In this context, we envisioned that connections exist between the accumulation of ^11^C-l-1MTrp in the MLNs and active responses to immunotherapies (online supplemental figure 4E), as a counter-regulatory mechanism to maintain the necessary immune privilege in the intestines that evolved for the perpetuation of the species in the ‘flaming’ body, and speculated that IDO1 expression in the MLNs could be an unprecedented off-tumor biomarker candidate for treatment outcomes in cancer immunotherapy. To test this assumption, we first performed competitive ^11^C-l-1MTrp PET imaging of B16F10-bearing mice treated with the IDO1 inhibitor INCB024360 as a blocking agent to demonstrate that ^11^C-l-1MTrp specifically binds to IDO1 in the MLNs. A significant decline in ^11^C-l-1MTrp uptake in the MLNs (5.12%±0.62% ID/g tissue) was observed in the mice treated with the inhibitor (figure 3E, F, online supplemental figure 4F, online supplemental table 2 and online supplemental video 2), supporting the conclusion that IDO1 is an unequivocal target of ^11^C-l-1MTrp in the MLNs.

10.1136/jitc-2021-002616.supp3 Supplementary data

**Figure 4:**
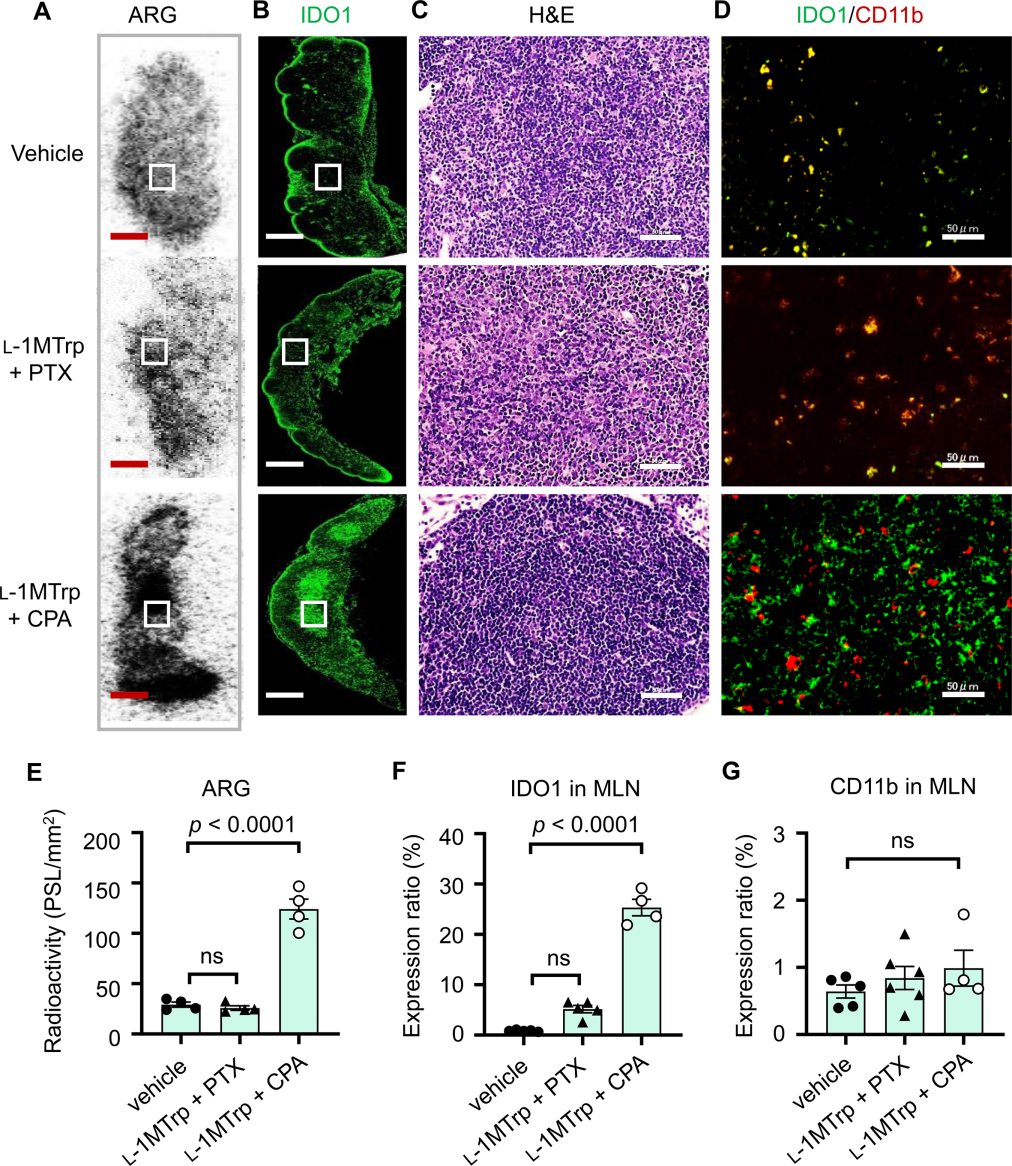
ARG and IDO1 expression in the MLNs. MLN samples were collected from melanoma-bearing mice treated with vehicle, l-1MTrp (orally, 5 g/kg feed) + CPA (150 mg/kg) or l-1MTrp (orally, 5 g/kg feed) + PTX (13.3 mg/kg) on day 23 after tumor inoculation. (A) The panel shows representative ex vivo ARG images of ^11^C-l-1MTrp binding in the MLNs. White rectangles show the area of interest. Scale bar: 1 mm. (B) The immunohistochemistry images show representative expression of the IDO1 protein in the MLNs. The green fluorescence signal shows the Alexa fluor 488-labeled anti-IDO1 antibody. Scale bar: 1 mm. (C) The panel shows H&E staining of MLN samples that were collected from melanoma-bearing mice, which was used for cell morphological investigation. Scale bar: 50 µm. (D) The images show staining for IDO1 (green) and CD11b (red). Scale bars: 50 µm. (E) Relative quantitative analysis of ex vivo ARG for ^11^C-l-1MTrp uptake in the MLNs was performed. Data represent the mean±SEM, n=4. (F, G) The protein expression of IDO1 (F) and CD11b (G) in the MLNs was quantified according to the data in panel D. Data represent the mean±SEM, n=4–6. All comparisons were performed using an unpaired two-tailed Student’s t-test. ARG, autoradiography; CPA, cyclophosphamide; IDO1, indoleamine-2,3-dioxygenase 1; MLN, mesenteric lymph node; PTX, paclitaxel.

Next, we sought to determine the spatial localization of ^11^C-l-1MTrp and IDO1 in the MLNs. We performed ex vivo autoradiography (ARG) and histopathology with MLN tissues harvested from treated mice described above. ARG of MLN sections indicated strong binding of ^11^C-l-1MTrp in the medulla of the MLNs of l-1MTrp+CPA-treated mice (figure 4A, E), which was consistent with the expression pattern of the IDO1 protein (figure 4B, F). In contrast, the IDO1 protein expression in the same zones of the MLNs was lower in samples from vehicle-treated and l-1MTrp+PTX-treated mice (figure 4B), resulting in poor tracer binding in those tissue samples (figure 4A, E). H&E staining of MLN sections did not reveal any noticeable tissue morphology variation (figure 4C), demonstrating that the treatment regimens had good biosafety. Although CD11b^+^ myeloid cells have been reported to inducibly express IDO1 in the tumor microenvironment,^27^ interestingly, double staining for IDO1 and CD11b revealed that CD11b^+^ cells in the MLNs of l-1MTrp+CPA-treated mice generally did not express IDO1 (figure 4D), indicating that IDO1 induction primarily occurred in CD11b-negative cells. Conversely, IDO1 and CD11b showed perfect colocalization in MLN sections from vehicle-treated and l-1MTrp+PTX-treated mice (figure 4D). The CD11b expression level exhibited undistinguishable differences in the MLNs among the three cohorts (figure 4G), further demonstrating a CD11b^+^ positive cell independent upregulation of IDO1 in MLNs. Overall, IDO1 expression in the MLNs is inducible in mice with IDO1 blockade-containing combinatorial immunotherapy and can be visualized by ^11^C-l-1MTrp PET.

### Longitudinal ^11^C-l-1MTrp imaging provides a new precision paradigm for combinatorial cancer immunotherapy

**Figure 5:**
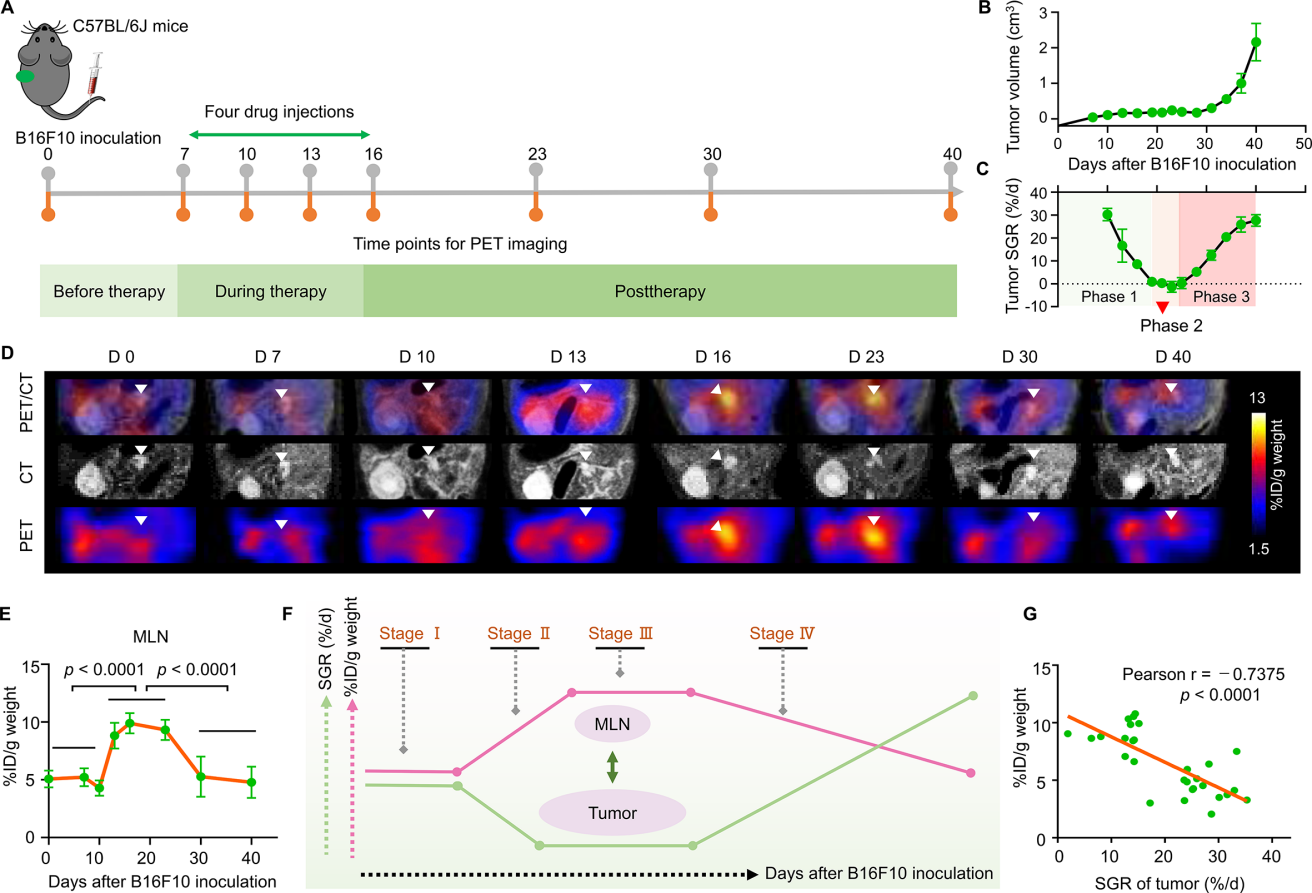
Longitudinal tracking of ^11^C-l-1MTrp in the MLNs following combinatorial cancer immunotherapy. (A) Schematic of the treatment plan and purpose of this study. A paradigm of precision medicine involving leveraging ^11^C-l-1MTrp PET imaging is proposed. Mice were inoculated with 5×10^4^ B16F10 cells in the left flank on day 0 and received combinatorial l-1MTrp (orally, 5 mg/kg feed) + CPA (intravenously, 150 mg/kg) therapy on days 7, 10, 13, and 16. After completing therapy, the mice were monitored for more than 24 days. ^11^C-l-1MTrp PET/CT was conducted on days 0, 7, 10, 13, 16, 23, 30, and 40. (B) Tumor growth curves. Data represent the mean±SEM, n=10. (C) Specific tumor growth rates. (D) Representative coregistered PET/CT images showing tracer uptake in the MLNs across the whole study period. PET images were summed from 60 to 75 min after ^11^C-l-1MTrp injection. White triangles indicate the MLNs. Each PET/CT image is representative of at least three independent experiments. (E) Quantification of the PET data for the MLNs. Each green dot represents one time point; for each time point, the number of mice ranged from 4 to 10. The orange line connects the mean of each set. Comparisons were performed using an unpaired two-tailed Student’s t-test. (F) Illustration of the relationship between the tumor SGR and tracer uptake in the MLNs. Four stages including tumor growth (stage I, day 0 to day 7), therapeutic activation (stage II, day 7 to day 16), therapeutic effect duration (stage III, day 16 to day 23), and therapeutic effect mitigation (stage IV, day 23 to day 40) were used to depict the status of tumors. The arrow indicates the trend in the signal in the MLNs. (G) Pearson correlation analysis of the tumor SGR and radioactive signal-quantified IDO1 within the MLNs. IDO1, indoleamine 2,3-dioxygenase 1; MLN, mesenteric lymph node; PET, positron emission tomography; SGR, specific growth rate.

Next, to unveil the inter-relationship between the dynamic IDO1 expression in the MLNs and the cancer-immune set point in individuals and to establish a PET imaging-guided precision medicine paradigm for cancer immunotherapy, we performed a longitudinal ^11^C-l-1MTrp PET imaging study throughout the entire treatment process in mice treated with l-1MTrp+CPA (figure 5A). Mice were subcutaneously injected with B16F10 tumor cells on day 0 and treated with l-1MTrp+CPA on days 7, 10, 13, and 16. Tumor growth was successfully inhibited until day 30 postinoculation but then increased from day 30 to day 40 (figure 5B). We employed the specific growth rate (SGR, the percentage change in tumor size per day, %/d) to define the real-time antitumor efficacy of the combinatorial immunotherapy. The SGR curves of the tumors revealed a three-phase process: phase I showed a gradual decline in the SGR that was attributed to the initiation of therapy and lasted until the end of the therapy. Although the therapeutic intervention was terminated on day 16, the therapeutic effect was sustained throughout phase II, which was characterized by inhibited tumor growth with a constant small SGR; in phase III, the therapeutic effect was gradually exhausted, resulting in tumor relapse, as demonstrated by the continuously increasing SGR (figure 5C).

^11^C-l-1MTrp PET imaging was carried out at multiple timepoints to closely monitor the therapeutic immune response (figure 5D). Before treatment, we determined the baseline uptake of ^11^C-l-1MTrp in the MLNs on days 0 (without tumor) and 7 (with a subcutaneous B16F10 tumor) and found that tracer uptake in the MLNs did not change depending on whether a tumor was engrafted. PET imaging showed that the tracer uptake in the MLNs on day 10 was similar to that on day 7, whereas the uptake increased drastically on day 13 and peaked on approximately day 16 (figure 5D, E); the percentage uptake on day 16 was double that at baseline (from ⁓5% to ⁓10% ID/g weight, figure 5E). The plateau in tracer uptake was maintained for approximately 1 week until day 23, even though the last drug injection was performed on day 16. Afterward, the tracer uptake in the MLNs showed a steep decline from day 23 to day 30 and finally demonstrated a relatively consistent decline to the baseline level from day 30 to day 40 (figure 5D, E).

We next assessed whether IDO1 expression in the MLNs is a predictor of the cancer-immune set point in the immunoediting process. As the tumor SGR is a real-time indicator of immunotherapeutic outcomes,^28^ we evaluated the relation between the percentage uptake of ^11^C-l-1MTrp in the MLNs and the tumor SGR (figure 5F). From the SGR curves (figure 5C), we divided the treatment process into four stages: tumor growth (I), therapeutic effect activation (II), therapeutic effect duration (III), and therapeutic effect mitigation (IV) (figure 5F). As expected, this tumor profile perfectly coincided with the percentage uptake of ^11^C-l-1MTrp in the MLNs: the ^11^C-l-1MTrp uptake increased when the therapeutic response was activated but decreased when the therapeutic effect was lost (figure 5F). Correlation analysis identified a significantly inverse association between the tumor SGR and percentage uptake in the MLNs (Pearson r=−0.7375, p<0.0001) (figure 5G). The unique ^11^C-l-1MTrp PET imaging patterns in MLNs of B16F10 mice (figure 5D) were further corroborated by ex vivo biodistribution studies (online supplemental figure 5 and online supplemental table 3), which show significantly higher uptake of ^11^C-l-1MTrp in MLNs at day 16 (29.34%±0.80% ID/g tissue) and day 23 (23.93%±4.03% ID/g tissue) postinoculation. These results indicated that ^11^C-l-1MTrp PET/CT could visually, quantitatively and spatiotemporally track the dynamic IDO1 expression in MLNs associated with immunotherapeutic efficacy and reveal the cancer-immune set point in individuals treated with IDO1 blockade-containing combinatorial immunotherapy.

### ^11^C-l-1MTrp imaging in the context of combinatorial immunotherapy with PD-1 and CTLA4 blockade

**Figure 6:**
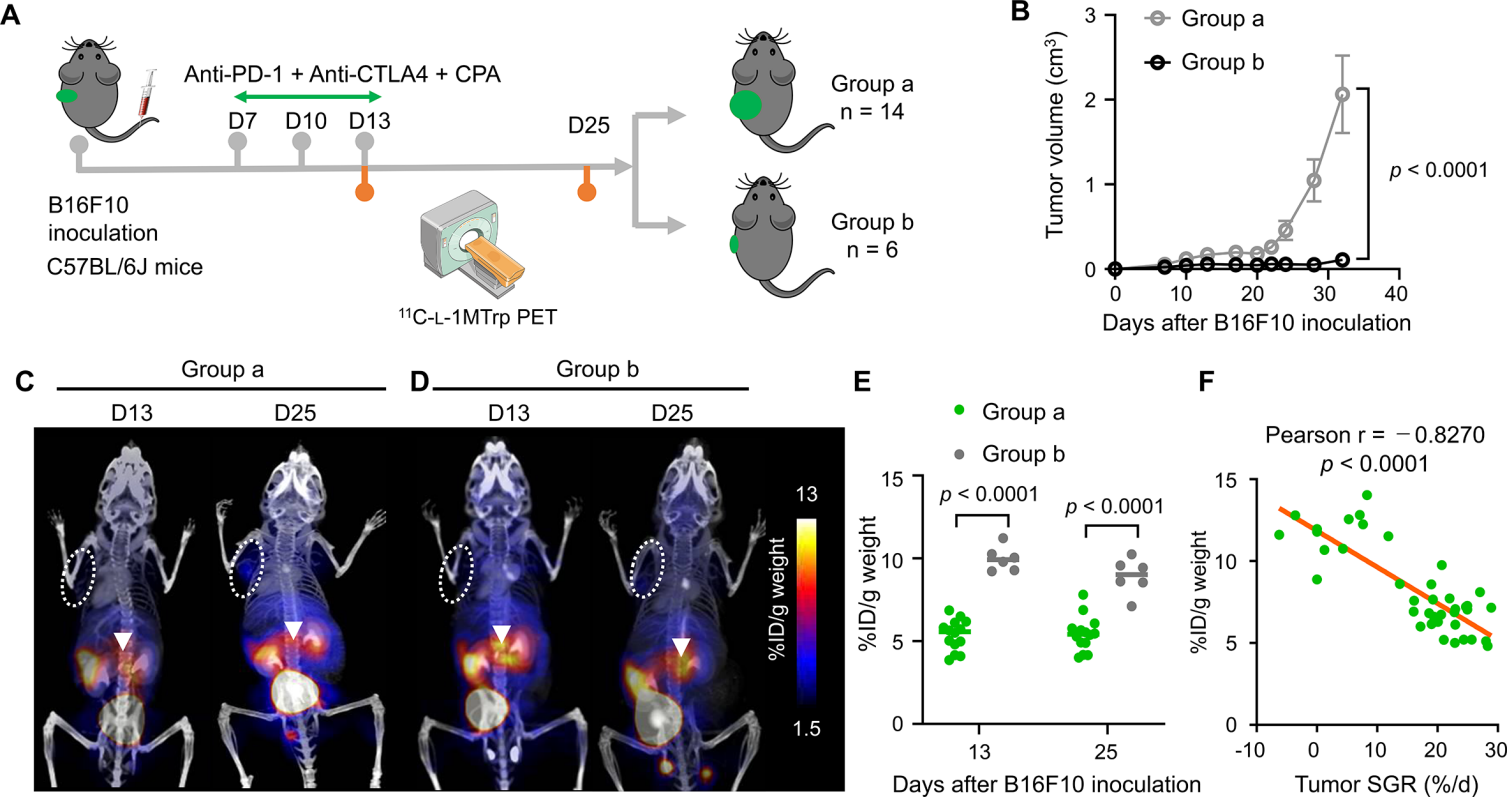
^11^C-l-1MTrp PET/CT monitoring of the therapeutic effect of a combinatorial anti-PD-1 + anti-CTLA4 + CPA immunotherapy regimen. (A) Treatment plan for C57BL/6J mice-bearing B16F10 tumors. B16F10 cells (5×10^4^) were inoculated into the left flank of mice on day 0; then, on days 7, 10 and 13, therapeutic antibodies (anti-PD-1 and anti-CTLA4) were administered via intraperitoneal injection, and CPA was injected via the tail vein. On day 32, the mice were divided into two groups according to tumor size, namely, groups a (n=14) and b (n=6). All mice were examined by ^11^C-l-1MTrp PET/CT on days 13 and 25. (B) Tumor growth curves for the mice in groups a and b. Data represent the mean±SEM, n=14 for group a and n=6 for group b. (C, D) Representative coregistered PET/CT images of mice in group a (C) and group b (D) acquired on days 13 and 25. ^11^C-l-1MTrp was injected intravenously. PET images were summed from 60 to 75 min after ^11^C-l-1MTrp injection. White circles indicate B16F10 tumors, while white triangles indicate the MLNs. (E) Quantification of the PET signal in the MLNs. Data represent the mean±SEM. n=14 for group a and n=6 for group b. (F) Pearson correlation analysis of the tumor SGR and radioactive signal-quantified IDO1 within the MLNs, n=40. All comparisons were performed by using two-way analysis of variance, followed by Bonferroni’s multiple comparisons post-test. CPA, cyclophosphamide; PD-1, programmed cell death 1; PET, positron emission tomography; MLN, mesenteric lymph node; SGR, specific growth rate.

To generalize ^11^C-l-1MTrp PET imaging for monitoring the cancer-immune status and antitumor response in mice receiving different immunotherapies, we evaluated another cohort of immunocompetent melanoma-bearing mice that received a triple-treatment regimen of anti-programmed cell death 1 (PD-1, clone RMP1-14, 10 mg/kg) + anti-cytotoxic T-lymphocyte-associated protein 4 (CTLA4, clone 9H10, 5 mg/kg) + CPA (150 mg/kg) (figure 6A). The poor responders and good responders were designated as group **a** (>0.5 cm^3^ tumor volume; n=14) and group **b** (<0.5 cm^3^ tumor volume; n=6), respectively, with the total cohort showing an overall positive response rate of 30% (6 of 20 mice) according to the tumor size measured on day 32. Group **a** had a significantly larger average tumor volume than group **b** on day 32 (2.08±0.35 cm^3^ and 0.40±0.14 cm^3^, respectively) (figure 6B and online supplemental figure 6A). The corresponding SGRs on day 32 were estimated to be 17.54%±1.75% /d in the poor responder group (n=14) and 2.99%±1.63% /d in the good responder group (n=6) (online supplemental figure 6B). As expected, ^11^C-l-1MTrp PET imaging on days 13 and 25 after tumor engraftment showed that group **a** exhibited low ^11^C-l-1MTrp uptake in the MLNs (figure 6C, E), whereas all mice in group **b** exhibited significantly higher tracer uptake in the MLNs (figure 6D, E), consistent with the generation of an effective antitumor response in the group **b** mice. In contrast, the tumor uptake exhibited little difference between the two groups (figure 6C, D). Furthermore, we evaluated the correlation between the tumor SGR and percentage uptake in the MLNs for B16F10-bearing mice in this treatment cohort. A strong negative correlation was observed between MLN radioactivity and the tumor SGR (figure 6F, Pearson r=−0.8270, p<0.0001). Collectively, these results suggest that ^11^C-l-1MTrp PET imaging can serve as a robust tool to gauge the cancer-immune set point and predict the antitumor immune efficacy of different types of immunotherapeutic blockade in addition to combination immunotherapy targeting multiple factors by monitoring IDO1 expression in the MLNs.

## Discussion

Recent clinical studies have aimed to identify appropriate methods to enable IDO1 regulation monitoring, as well as to determine the cancer-immune status and predict the therapeutic efficacy of IDO1-based combinatorial immunotherapies. Quantitative PET imaging is an ideal tool for these purposes. We first assessed ^11^C-l-1MTrp in mice bearing contralateral human tumors with distinct IDO1 expression patterns. Then, we applied ^11^C-l-1MTrp to longitudinally monitor whole-body IDO1 variations in immunocompetent melanoma-bearing mice treated with l-1MTrp plus either chemotherapeutic drugs or antibodies targeting PD-1 and CTLA4. Unexpectedly, we observed pronounced variations in the ^11^C-l-1MTrp signal in the MLNs rather than the tumors, corresponding to effective immunoediting. Moreover, the PET signals in the MLNs closely correlated with therapeutic efficacy, implying that the IDO1 status in the MLNs is an unprecedented surrogate marker of the cancer-immune set point for combinatorial immunotherapies.

IDO1 is a member of the immune checkpoint family, exhibiting immunosuppressive effect similar to PD-L1, a Food and Drug Administration-approved biomarker for predicting the efficiency of anti-PD-1/PD-L1 checkpoint therapies.^29^ The success of PD-L1-based strategies spurred us to identify a method to visualize IDO1 expression and help improve the efficacy of IDO1-targeted immunotherapy. In our study, we applied the tracer ^11^C-l-1MTrp, which is capable of detecting IDO1 with good specificity and sensitivity, and performed target validation. Further application of ^11^C-l-1MTrp to monitor therapeutic efficacy in mice treated with one of two IDO1 blockade + chemotherapy regimens showed little ability to distinguish between good and poor responders via tumor PET imaging (figures 2 and 3A); a similar phenomenon was reported in a previous study.^30^

Unexpectedly, we identified the MLNs as a robust off-tumor IDO1 site for active immune response monitoring (figures 3 and 4), different from currently known methods that focus on tumor sites (online supplemental table 4).^31–44^ These findings enabled us to distinguish l-1MTrp+CPA as a more effective regimen than l-1MTrp+PTX for treating murine B16F10 melanoma (figure 3), simplifying the assessment of immunotherapy effectiveness, and allowing oncologists to rapidly analyze and screen more effective combination regimens on a common platform. Although various studies have indicated that the combination of an IDO1 inhibitor with chemotherapy induces antitumor immunity, which leads to immune-mediated clearance of IDO1-expressing and nonexpressing poorly immunogenic tumors, including melanoma,^1–3 11 19^ failed clinical trials with IDO1 inhibitors have set back the development of this approach.^9^ To overcome this limitation in clinical translation, IDO1 PET imaging methods, reproducible in situ measurement of off-tumor IDO1 dynamics to visualize the immunoediting process during cancer therapy, may offer a solution both in clinical and research settings, which would speed up the development on combination regimens with an extremely high efficiency, further elucidate drug mechanisms of action, and help tailor rational therapy regimens.^45 46^

PET imaging has become an essential part of precision medicine and facilitates the rapid development of personalized treatment.^47^ Successful precision therapy is based on objective individual stratification and the identification of ongoing therapeutic responses.^48^ To examine whether IDO1 PET imaging of the MLNs can achieve these goals, we performed longitudinal and multidimensional tracking of the cancer-immune set point in melanoma-bearing mice treated with an effective regimen of l-1MTrp+CPA, and a significant positive relationship was identified between ^11^C-l-1MTrp uptake in the MLNs and therapeutic response. These results demonstrated that IDO1 expression in the MLNs can serve as a biomarker of the cancer-immune set point in immunoediting induced by IDO1 blockade-containing combinatorial immunotherapy (figure 5). Moreover, the experimental results of triple treatments with PD-1 blockade + CTLA4 blockade + CPA in a non-IDO1-targeted combinatorial regimen suggest that the IDO1 PET imaging approach has the potential to be used for response prediction and individual stratification. These results indicate that IDO1 PET imaging in the MLNs is a promising approach to create a precision paradigm for multiple combinatorial immunotherapies.^49^

Of note, our study also had some limitations. In particular, the underlying mechanisms of IDO1 in the MLNs responding to active immune responses are unknown. A decisive illustration of the relationship between IDO1 in the MLNs and the immune response might strengthen our understanding of insights into IDO1-targeted immunotherapy and clarify the clinical value of this imaging paradigm. Following this proof-of-concept study, further exploration of the exact mechanism of IDO1 in MLNs during immunotherapy and the geniune clinical values of IDO1 PET imaging is ongoing.

## Conclusion

In conclusion, we have demonstrated a quantitative imaging method capable of specifically detecting dynamic IDO1 expression and monitoring the cancer-immune set point without the limitations associated with biopsy. Additionally, we established a simple method for the multidimensional measurement of IDO1 regulation during immunotherapy in animal models. Given the prior approval and good safety profile of l-1MTrp in conjunction with the wide availability of the ^11^C-l-1MTrp radiotrace, as well as the commonplace use of PET imaging, evaluation of this technology in humans is expected, with efforts already underway. Collectively, PET imaging of IDO1 in the MLNs holds potential as a useful addition to understanding the gains and losses of IDO1 regimens and improving therapeutic outcomes by designing rigorous clinical trials. The paradigm would also provide a robust tool for fast assessment of the efficacy of IDO1-involved in regimens of precision cancer immunotherapies.

## Data Availability

All data relevant to the study are included in the article or uploaded as supplementary information.
