# Supplementary material for: Off-tumor IDO1 target engagements determine the cancer-immune set point and predict the immunotherapeutic efficacy
Source: J Immunother Cancer. 2021 Jun 20;9(6):e002616. doi: 10.1136/jitc-2021-002616 (PMC8237741; doi:10.1136/jitc-2021-002616)
Supplement: Supplementary data [file jitc-2021-002616supp001.pdf]

Supporting Information for:

Off-tumor IDO1 target engagements determine the cancer-immune set point and predict the immunotherapeutic efficacy

Authors: Lin Xie[1#], Kuan Hu[1#], Yanhong Duo[2#], Takashi Shimokawa[3], Katsushi Kumata[1], Yiding Zhang[1], Cuiping Jiang[1], Lulu Zhang[1], Nobuki Nengaki[1], Hidekatsu Wakizaka[1], Yihai Cao[2*], Ming-Rong Zhang[1*]

Affiliations:
[1]Department of Advanced Nuclear Medicine Sciences, National Institute of Radiological Sciences, National Institutes for Quantum and Radiological Science and Technology, 4-9-1 Anagawa, Inage-ku, Chiba 263-8555, Japan.
[2]Department of Microbiology, Tumor and Cell Biology, Karolinska Institute, 171 77, Stockholm, Sweden
[3]Department of Accelerator and Medical Physics, National Institute of Radiological Sciences, National Institutes for Quantum and Radiological Science and Technology, 4-9-1 Anagawa, Inage-ku, Chiba 263-8555, Japan.

***To whom correspondence should be addressed:**
Yihai Cao, M.D., PhD.
Department of Microbiology, Tumor and Cell Biology, Karolinska Institute, 171 77 Stockholm, Sweden. Phone: +46705597596; E-mail: yihai.cao@ki.se.
Ming-Rong Zhang, PhD.
Department of Advanced Nuclear Medicine Sciences, National Institute of Radiological Sciences, National Institutes for Quantum and Radiological Science and Technology, 4-9-1 Anagawa, Inage-ku, Chiba 263-8555, Japan. Phone: +81-43-382-3709; Fax: +81-43-206-3261; E-mail: zhang.ming-rong@qst.go.jp

Xie L, *et al. J Immunother Cancer* 2021; 9:e002616. doi: 10.1136/jitc-2021-002616

Supplementary Methods

Chemicals, antibodies, cell lines and animals

L-1MTrp was purchased from Sigma-Aldrich (St. Louis, MO, USA). CPA was acquired from Tokyo Chemical Industry (Tokyo, Japan). PTX was acquired from Sigma-Aldrich. Therapeutic anti-PD1 (clone RMP1-14) and anti-CTLA4 (clone 9H10) monoclonal antibodies were purchased from BioLegend (London, UK). The human tumor cell lines NCI-H69 and MDA-MB231 and the mouse melanoma cell line B16F10 were purchased from the American Type Culture Collection (Manassas, VA, USA). NCI-H69 cells were grown in RPMI-1640 medium supplemented with 10% fetal bovine serum, penicillin (100 U/mL), and streptomycin (0.1 mg/mL). MDA-MB231 and B16F10 cells were maintained and passaged in DMEM supplemented with 10% fetal bovine serum, penicillin (100 U/mL), and streptomycin (0.1 mg/mL) in an atmosphere of 5% CO_2 at 37°C.

qRT-PCR analysis

RNA was extracted from tumor cells and tumor tissues using the RNeasy Mini Kit (Qiagen, Valencia, CA, USA) according to the manufacturer's protocol. The quality of the total RNA was verified using the 260/280 nm ratio with a NanoDrop 2000 (Thermo Scientific, Wilmington, DE, USA). qRT-PCR was performed using a TaqMan system on an Applied Biosystems StepOne™ machine (Carlsbad, CA, USA) according to the manufacturer's instructions. Target-specific primers and probes for human *IDO1 (Hs00984148_m1),* mouse *IFN-γ (Mm01168134_m1),* mouse

Stat1(Mm01257286_m1), mouse *GranzymeB* (*Mm00442837_m1*) and 18S *ribosomal RNA* (*18S rRNA*, *Hs99999901_s1*) were purchased from Applied Biosystems. The normalized Ct value of each gene was obtained by subtracting the Ct value for *18S rRNA*. The fold change of each gene in mRNA levels versus the corresponding controls level was calculated.

Tumor treatment regimens

Animals were randomly assigned to various test groups in a blinded manner. To ensure stable plasma Trp and other amino acid levels during the study, mice were fasted overnight with free water access prior to all radioactivity uptake experiments. The individuals handling the animals and conducting animal therapies were blinded to the experimental design.

Mouse syngeneic tumor model and treatment regimens: Syngeneic tumor models were established in immunocompetent male C57BL/6J mice (Japan SLC, Shizuoka, Japan), via subcutaneous injection of 5×10^4 B16F10 melanoma cells into the flank in a total volume of 0.1 mL serum-free medium. For IDO1 blockade-containing combinatorial immunotherapy, seven days after tumor implantation, mice were divided randomly into three groups: L-1MTrp plus CPA group, the L-1MTrp plus PTX group, and the vehicle group. L-1MTrp was orally administered using a powder feed system to achieve L-1MTrp infusion in a nearly continuous manner. The powder feed system contained L-1MTrp added to a powder diet (Oriental Yeast, Tokyo) at 5 g/kg feed, which

was thoroughly mixed into the daily feed ration. Mice ate 3.5–3.7 g/day, similar to the dietary consumption without the drug. L-1MTrp was administered in the feed for ten days, starting on day 7 after tumor implantation, with freshly prepared L-1MTrp feed given every other day. CPA (150 mg/kg per injection) or PTX (13.3 mg/kg per injection) was administered intravenously on days 7, 10, 13, and 16. The mice in the vehicle group received a corresponding dose of vehicle and placebo powder diet without the active ingredient. To examine the character of ^{11}C-L-1MTrp PET/CT imaging in the different treatment outcomes among the three treatment strategies, we selected these 23 day-mice after implantation as the imaging subjects, who were quiescent after approximately 1 week following all treatment stimulations. For determining the interrelationship between the dynamic IDO1 expression in the MLNs and the cancer-immune set point in individuals, a longitudinal ^{11}C- L-1MTrp PET/CT imaging and distribution studies were performed on days 0, 7, 10, 13, 16, 23, 30, and 40 after tumor inoculation throughout the entire treatment process in mice treated with L-1MTrp + CPA. Mice were humanely sacrificed using isoflurane anesthesia when they were moribund or at the experimental endpoint of 40 days after tumor implantation.

In the anti-PD-1 and anti-CTLA4 dual-blockade experiment, male C57BL/6J mice were inoculated subcutaneously with 5×10^4 B16F10 cells into the flank in a total volume of 0.1 mL serum-free medium. On days 7, 10, and 13 after tumor inoculation, 10 mg/kg anti-PD-1 plus 5 mg/kg anti-CTLA4 therapeutic antibodies were mixed in a single injection and administered via

intraperitoneal injection combined with intravenous injection of 150 mg/kg CPA. To generalize ^{11}C-L-1MTrp PET imaging for monitoring the cancer-immune set point and antitumor response in mice receiving different immunotherapies, on day 13 and 25 after tumor transplantation, all mice received a triple-treatment regimen of anti-PD-1 + anti-CTLA4 + CPA were used for imaging study after ^{11}C-L-1MTrp injection. At the end of the study, the mice were segregated into two groups termed group a (Tumor volume more than 0.5 cm^3 represents the poor responder) and group b (Tumor volume less than 0.5 cm^3 represents the good responder) on day 32 after tumor inoculation.

Tumor response measurement

Tumor response was monitored every 2–3 days by measuring tumor volume (V) using calipers. The formula V = (length × width^2) × 0.5 was used to estimate tumor volume, and results are presented in cm^3. To objectively describe the real-time antitumor response during the posttreatment follow-up period, the volumetric growth rate of each tumor per day was quantified by the SGR, the percentage volume change per day in measurement intervals, using the formula $SGR = \ln (V_1 / V_0) / (t_1 - t_0) \times 100\%$, where V_0 is the tumor volume at the start of measurement (t_0), and V_1 is the volume at the end of this period (t_1).

Dynamic PET/CT and PET data analysis

Blocking experiments were performed by intravenous co-injection an excess of unlabeled IDO1 inhibitor INCB024360 (10 mg/kg) or L-1MTrp (50 mg/kg) with the radiotracer. All list-mode acquisition data were sorted into three-dimensional sinograms, which were then Fourier-rebinned into two-dimensional sonograms, and corrected for scanner dead time, randoms, and decay of the injected radiotracer. Dynamic images were reconstructed with filtered back-projection using Hanning's filter and a Nyquist cut-off of 0.5 cycles/pixel. Regions of interest (ROIs) in tumors and tissues were drawn using Siemens Inveon Research Workplace (IRW) 4.0 software. The average radioactivity concentration was obtained from the mean pixel values in the ROI volume, which was manually positioned based on the tumor and tissue contours in the orthogonal plane with the largest diameter. Regional uptake of radioactivity was decay-corrected to injection time, normalized to the whole bodyweight of a mouse, and expressed as %ID/g weight. Time activity curves (TACs) of ^{11}C-L-1MTrp were determined.

To provide the anatomical landscape of PET images, after PET scans, contrast-enhanced CT scans for the same mice were immediately performed after an injection of 0.4 mL nonionic contrast medium (Iopamiron 370, Bayer, Osaka, Japan) using a small-animal CT system (R_mCT2; Rigaku, Tokyo). The scan conditions included the radiation parameters 200 μA and 90 kV, an FOV of 60 mm, and an acquisition time of 34 s. CT images were collected, reconstructed, and observed using I-View-R software (Rigaku, The Woodlands, TX, USA). Averaged CT attenuation images and

dynamic PET images acquired 60–75 min after injection were reconstructed and fused using Siemens IRW 4.0 software.

Ex vivo autoradiography

Sixty min after an intravenous injection of ^{11}C-L-1MTrp (1.7–1.9 MBq/0.1 mL), mice were sacrificed by cervical dislocation. The MLNs were harvested immediately, placed in direct contact with a BAS-MS 2325 imaging plate (Fujifilm, Tokyo) for 30 min, and analyzed using the BAS 5000 Bio Imaging Analyzer System with Multi Gauge software v.2.3 (Fujifilm). The amount of radioactivity in the MLNs was quantified and normalized, and results are expressed as photostimulated luminescence per unit area (PSL/mm^2).

Histopathology

Mouse samples were collected, fixed in 10% formalin and embedded in paraffin. H&E staining of tissue sections from treated and vehicle-group mice was performed according to standard procedures. To assay IDO1 expression and distribution, double immunofluorescence staining was performed on single serial sections (5μm) using the OPAL 3-Plex Kit (PerkinElmer, Waltham, MA, USA) according to the manufacturer's instructions. The tissue sections were deparaffinized in xylol and rehydrated in graded alcohol. Antigen retrieval was performed at 95 °C for 10 minutes in 10

mM citrate buffer (pH = 6). The primary antibodies rabbit anti-human IDO1 (1:100; EPR20374, Abcam, Cambridge, UK) or rat anti-mouse IDO1 (1:100; sc-53978, Santa Cruz Biotechnology, Milan, Italy) with rabbit anti-mouse CD11b (1:100; ab52478, Abcam) were incubated with the tissue sections on slides for 60 minutes at room temperature. Secondary antibodies recognizing the anti-human IDO1, anti-mouse IDO1 and anti-mouse CD11b antibodies were labeled with Alexa Fluor® 488 goat anti-rabbit IgG (1:500; A27034, Invitrogen, Carlsbad, CA), Alexa Fluor® 488 goat anti-rat IgG (1:500; A-11006, Invitrogen) and Alexa Fluor®546 goat anti-rabbit IgG (1:500; A-11035, Invitrogen), respectively. Images were acquired using a Keyence BZ-X710 microscope (Keyence, Osaka, Japan). The frequency of positively stained areas was measured automatically by using specialized Hybrid Cell Count software (Keyence), and the results are expressed as the percentage of the total tissue area exhibiting positive staining. Negative control slides were processed in the absence of primary antibody, secondary antibody, or with isotype control IgG to ensure specificity.

Statistical analysis

Data were analyzed using Prism version 8.0 software (GraphPad Software, La Jolla, CA). Comparisons among groups were performed using two-way ANOVA with Bonferroni's multiple comparisons posttest or unpaired two-tailed Student's t-test. Data are presented as the mean ± the

standard error of the mean (s.e.m). The threshold for statistical significance was set as $p < 0.05$. Pearson's correlation analysis was used to estimate the relationship between radioactivity and the SGR of tumors by using the percentage uptake in the MLNs as a dependent variable and the tumor SRG as an independent variable indicator.

Supplementary Figures

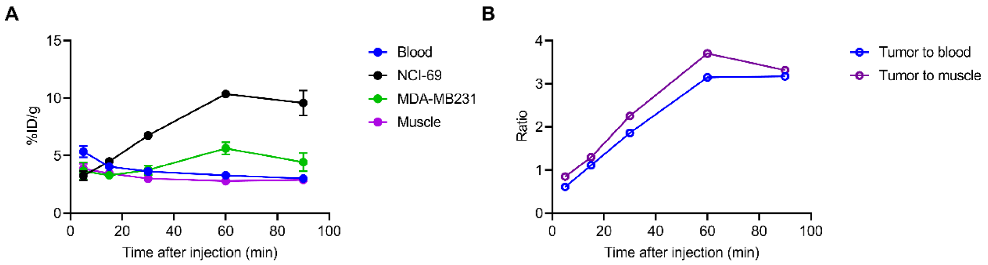

Supplementary Fig. 1 Dynamic changes of ^{11}C-L-1MTrp in selected organs (A) *Ex vivo* biodistribution analysis of the activity of ^{11}C-L-1MTrp in the blood, NCI-H69 tumor, MDA-MB231 tumor, and muscle at different time points; mean ± s.e.m., n = 3. (**B**) Changes in the tumor (%ID/g in NCI-69 tumors)-to-blood- and tumor-to-muscle ratios of ^{11}C- L-1MTrp at specific time points post tracer injection. Each dot represents the mean of three mice.

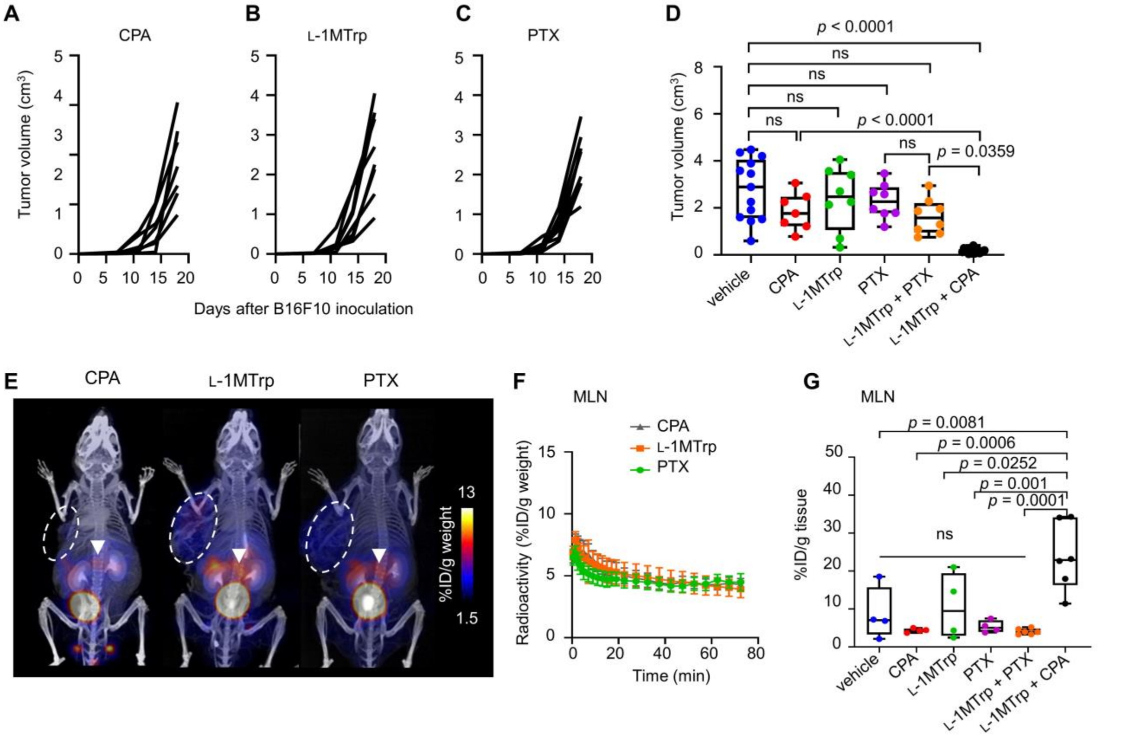

Supplementary Fig. 2 Antitumor effects of monotherapies and the corresponding [11]C- L-1MTrp PET/CT imaging. (A-C) Individual tumor growth curves of mice treated with CPA (150 mg/kg), L-1MTrp (orally, 5 g/kg feed), PTX (13.3 mg/kg). (D) Tumor volume of mice on day 18 postinoculation. Data represent the mean ± s.e.m, n = 13 for vehicle group, n = 7 for CPA group, n = 8 for L-1MTrp, n = 8 for PTX group, n = 8 for L-1MTrp + PTX group, and n = 16 for L-1MTrp + CPA group. (E) Representative PET/CT images of melanoma-bearing mice after treatment with CPA, L-1MTrp, or PTX only. Imaging was performed on day 23 after B16F10 cell inoculation, and ^{11}C-L-1MTrp was injected intravenously. PET images were summed from 60 to 75 min postinjection. White circles indicate B16F10 tumors, and white triangles indicate the MLNs. Each PET/CT image is representative of at least 3 independent experiments. (F) Time-activity curves showing the dynamics of ^{11}C-L-1MTrp in the MLNs of three groups of mice. (G) *Ex vivo* measurement of ^{11}C-L-1MTrp in the MLNs of treated tumor-bearing mice at 60 min postinjection. Data represent the mean ± s.e.m, n = 4–6 mice.

Xie L, *et al. J Immunother Cancer* 2021; 9:e002616. doi: 10.1136/jitc-2021-002616

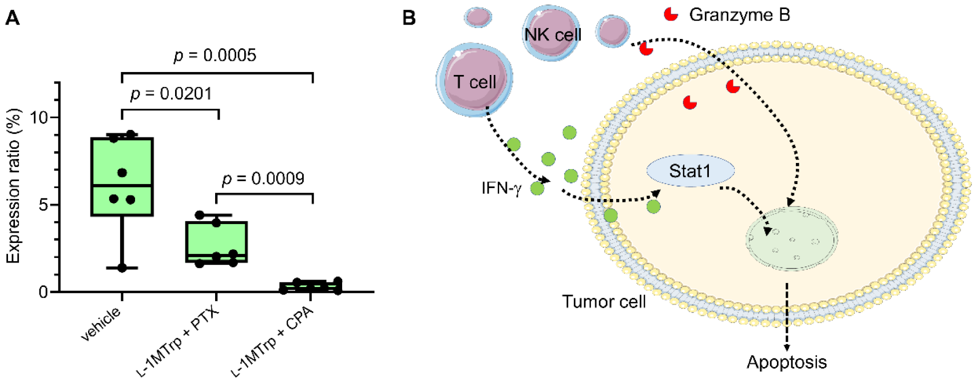

Supplementary Fig. 3 (A) IDO1 protein expression ratio in B16F10 tumor tissues acquired from mice on day 23 after treatment with vehicle, L-1MTrp + PTX, or L-1MTrp + CPA determined by microscopy. Data represent mean ± s.e.m, n = 5. Comparisons were performed using an unpaired two-tailed Student's t-test. **(B)** Schematic of the potential mechanism by which the regimen L-1MTrp + CPA activates the antitumor response to kill cancer cells. It is proposed that L-1MTrp + CPA treatment induced IFN-γ release from T and NK cell in the tumor microenvironment, which further resulted in Stat1 upregulation, as a result, leading to the apoptosis of tumor cells. On the other hand, the T and NK cells can secret granzyme B responding to L-1MTrp + CPA treatment, which co-promoted the tumor cell apoptosis.

Xie L, *et al. J Immunother Cancer* 2021; 9:e002616. doi: 10.1136/jitc-2021-002616

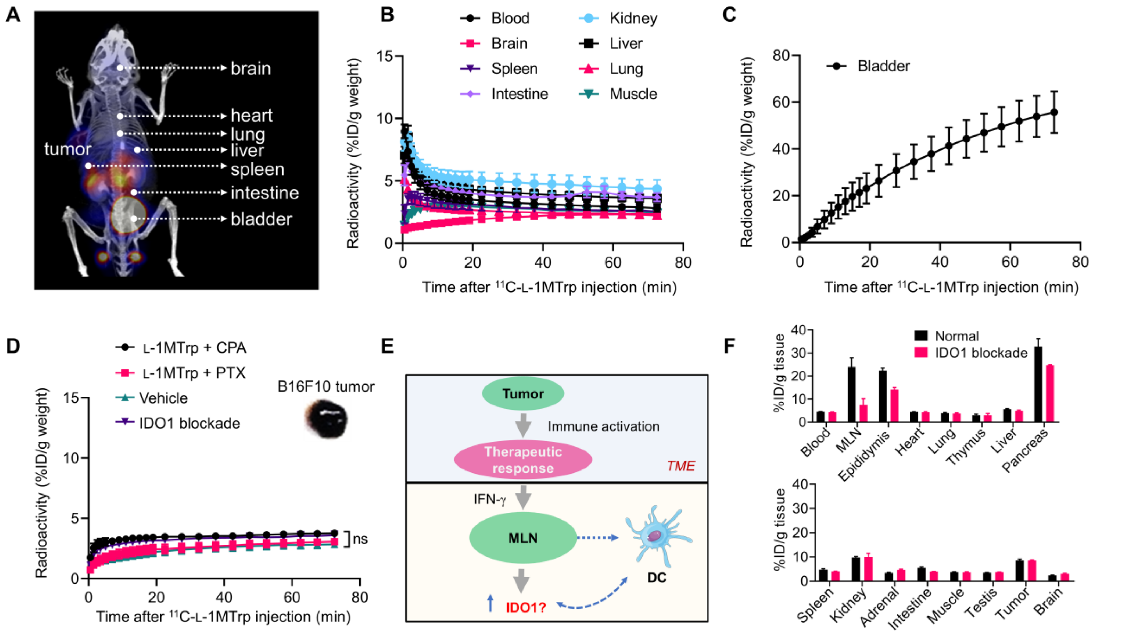

Supplementary Fig. 4 Quantification of ^{11}C-L-1MTrp signal in different organs from the PET images and ex vivo biodistribution of the radiotrace in major organs. (A) Representative ^{11}C-L-1MTrp PET/CT image of a mouse treated with L-1MTrp (orally, 5 g/kg) + CPA (150 mg/kg). The major organs are labeled. **(B)** Time-activity curves of ^{11}C-L-1MTrp in selected organs of mice treated with L-1MTrp + CPA (mean ± s.e.m., n = 4). (**C**) The time-activity curve of ^{11}C-L-1MTrp in bladder in treated mice (mean ± s.e.m., n = 4). (**D**) Time-activity curves of ^{11}C-L-1MTrp in B16F10 tumors in treated mice. Data represent mean ± s.e.m., n = 4. (**E**) Schematic showing the connections between the antitumor immune response in the tumor microenvironment (TME) and IDO1 expression in the MLNs. Therapeutic response elicited by drug treatments restores the antitumor response in tumor microenvironment. As an indicator of the intensity of antitumor response, IFN-γ can induce the upregulation of IDO1 in MLNs, which inversely reflects the cancer-immune status in TME. (**F**) Changes in ^{11}C-L-1MTrp uptake in B16F10 tumors, MLNs, and major tissues after INCB024360 blockade. The uptake of ^{11}C-L-1MTrp in the tissues was determined by *ex vivo* biodistribution analysis at 60 min postinjection. Data show the mean ± s.e.m., n = 5 for the normal group and n = 4 for the blockade group. All comparisons were performed using an unpaired two-tailed Student's t-test.

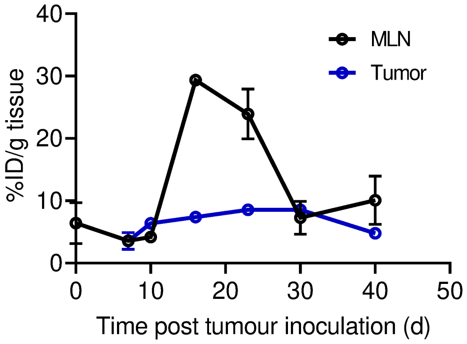

Supplementary Fig. 5 Quantification of ^{11}C-L-1MTrp uptake in the MLNs and tumors at different time points post tumor inoculation determined from *ex vivo* biodistribution analysis. Data represent mean ± s.e.m., n = 4-6.

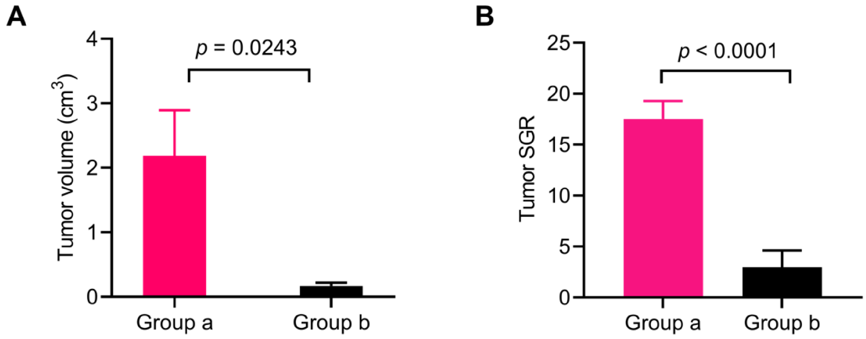

Supplementary Fig. 6 Tumor volume and SGR of B16F10-bearing mice treated with triple therapeutic regimen. (**A**) Mean tumor volumes in group a and group b at day 32 post tumor inoculation. (**B**) Specific tumor growth rates in group a and group b. Data represent the mean ± s.e.m., n = 14 for the group a and n = 6 for the group b. All comparisons were performed using an unpaired two-tailed Student's t-test.

Supplementary Tables

Supplementary Table 1. Biodistribution characterization of ^{11}C-L-1MTrp uptake in immunodeficient mice bearing human tumors. *Ex vivo* biodistribution data for ^{11}C-L-1MTrp uptake in immunodeficient BALB/c nude mice bearing s.c. NCI-H69 and MDA-MB231 tumors; data were collected at 5, 15, 30, 60, and 90 min after radioinjection. Data are expressed as the mean %ID/g tissue ± standard error of the mean (s.e.m.; n = 3).

Tissue	5 min	15 min	30 min	60 min	90 min
Blood	5.35 ± 0.49	4.05 ± 0.09	3.63 ± 0.12	3.29 ± 0.16	3.02 ± 0.11
NCI-H69 tumor	3.26 ± 0.42	4.49 ± 0.06	6.75 ± 0.38	10.36 ± 0.16	9.58 ± 1.08
MDA-MB231 tumor	3.66 ± 0.75	3.30 ± 0.34	3.75 ± 0.40	5.63 ± 0.52	4.43 ± 0.79
Heart	4.98 ± 0.66	3.70 ± 0.24	3.16 ± 0.05	3.01 ± 0.12	3.05 ± 0.05
Lung	4.03 ± 0.66	3.59 ± 0.19	3.19 ± 0.20	2.71 ± 0.14	2.89 ± 0.13
Liver	5.70 ± 0.48	4.69 ± 0.20	4.38 ± 0.13	3.81 ± 0.02	3.60 ± 0.18
Pancreas	37.88 ± 2.24	42.22 ± 1.80	42.54 ± 1.29	36.09 ± 2.26	34.97 ± 3.85
Spleen	5.28 ± 0.60	4.38 ± 0.14	3.82 ± 0.14	3.17 ± 0.13	3.59 ± 0.17
Kidney	7.79 ± 0.61	5.95 ± 0.13	5.86 ± 0.26	5.00 ± 0.30	5.18 ± 0.23
Adrenal gland	3.42 ± 0.87	2.36 ± 0.50	1.61 ± 0.19	1.91 ± 0.37	2.93 ± 0.30
Intestine	4.01 ± 0.28	3.60 ± 0.11	3.88 ± 0.08	3.81 ± 0.28	4.27 ± 0.34
Muscle	3.85 ± 0.47	3.47 ± 0.12	3.10 ± 0.17	2.80 ± 0.09	2.89 ± 0.11
Brain	1.77 ± 0.18	2.14 ± 0.11	2.59 ± 0.10	2.13 ± 0.06	2.15 ± 0.38

Supplementary Table 2. Biodistribution characterization of ^{11}C-L-1MTrp uptake in B16F10 tumor-bearing immunocompetent mice treated with IDO1 blockade-containing combinatorial immunotherapies or monotherapies. *Ex vivo* biodistribution data for ^{11}C-L-1MTrp uptake collected at 60 min after ^{11}C-L-1MTrp injection in six cohorts: vehicle, L-1MTrp, CPA, PTX, L-1MTrp + PTX, and L-1MTrp + CPA. A competition study was performed in L-1MTrp + CPA treatment mice by co-injection the "cold" IDO1 inhibitor INCB024360 (10 mg/kg) with ^{11}C-L-1MTrp. Data are expressed as the mean %ID/g tissue ± standard error of the mean (s.e.m.; n = 4-6).

Organ	Vehicle	L-1MTrp	CPA	PTX	L-1MTrp + PTX	L-1MTrp + CPA	L-1MTrp + CPA + INCB024360
Blood	3.03 ± 0.21	3.45 ± 0.37	4.40 ± 0.11	4.28 ± 0.19	3.63 ± 0.25	4.43 ± 0.16	4.19 ± 0.23
**MLN**	**7.52 ± 3.86**	**10.62 ± 4.38**	**4.38 ± 0.37**	**5.35 ± 0.80**	**4.07 ± 0.31**	**23.93 ± 4.03**	**7.39 ± 2.78**
Epididymis	9.52 ± 1.09	11.28 ± 1.10	24.67 ± 0.79	18.25 ± 1.20	14.42 ± 1.01	22.44 ± 1.05	14.19 ± 0.83
Heart	3.08 ± 0.21	3.82 ± 0.42	4.42 ± 0.16	4.43 ± 0.21	3.70 ± 0.22	4.37 ± 0.16	4.23 ± 0.31
Lung	2.86 ± 0.23	3.65 ± 0.43	3.85 ± 0.18	3.98 ± 0.20	3.31 ± 0.21	3.95 ± 0.15	3.71 ± 0.26
Thymus	2.76 ± 0.20	3.34 ± 0.41	3.94 ± 0.26	3.75 ± 0.41	3.18 ± 0.19	3.06 ± 0.41	2.98 ± 0.74
Liver	3.94 ± 0.28	4.10 ± 0.47	5.21 ± 0.12	5.42 ± 0.29	4.14 ± 0.44	5.66 ± 0.25	4.96 ± 0.32
Pancreas	25.78 ± 4.9	28.49 ± 2.63	28.23 ± 0.99	25.36 ± 1.25	20.81 ± 3.58	32.86 ± 3.43	24.68 ± 0.33
Spleen	3.15 ± 0.20	3.74 ± 0.34	4.26 ± 0.24	4.62 ± 0.39	3.57 ± 0.22	4.76 ± 0.38	3.99 ± 0.14
Kidney	7.23 ± 0.72	7.75 ± 0.94	10.26 ± 0.52	8.38 ± 0.28	6.92 ± 0.55	9.74 ± 0.44	9.98 ± 1.51
Adrenal gland	2.55 ± 0.49	2.21 ± 0.44	5.01 ± 0.30	3.58 ± 0.66	3.43 ± 0.30	3.36 ± 0.20	4.68 ± 0.26
Intestine	4.62 ± 0.44	3.94 ± 0.48	4.94 ± 0.42	4.82 ± 0.38	3.66 ± 0.45	5.55 ± 0.36	3.89 ± 0.08
Muscle	2.56 ± 0.17	3.09 ± 0.27	3.61 ± 0.21	3.86 ± 0.17	3.17 ± 0.18	3.71 ± 0.13	3.61 ± 0.25
Testis	2.17 ± 0.08	2.07 ± 0.11	3.84 ± 0.14	2.87 ± 0.13	4.51 ± 0.58	3.46 ± 0.07	3.66 ± 0.08
Tumor	6.43 ± 0.63	4.72 ± 0.75	7.14 ± 1.07	6.71 ± 0.50	6.03 ± 0.31	8.55 ± 0.56	8.54 ± 0.25
Brain	2.17 ± 0.22	1.84 ± 0.25	3.12 ± 0.14	1.50 ± 0.04	2.02 ± 0.16	2.44 ± 0.11	3.07 ± 0.14

Xie L, *et al. J Immunother Cancer* 2021; 9:e002616. doi: 10.1136/jitc-2021-002616

Supplementary Table 3. Longitudinal evaluation of the ^{11}C-L-1MTrp biodistribution in B16F10 tumor-bearing immunocompetent mice treated with IDO1 blockade-containing combinatorial immunotherapy. *Ex vivo* biodistribution data of ^{11}C-L-1MTrp were collected at 60 min after ^{11}C-L-1MTrp injection over the course of combinatorial therapy with L-1MTrp plus CPA. Data are expressed as the mean %ID/g tissue ± standard error of the mean (s.e.m.; n = 4-6).

Organ	D 0	D 7	D 10	D 16	D 23	D 30	D 40
Blood	4.61 ± 0.11	4.08 ± 0.36	4.38 ± 0.12	4.95 ± 0.33	4.43 ± 0.16	3.97 ± 0.09	3.07 ± 0.28
**MLN**	**6.41 ± 3.27**	**3.58 ± 0.52**	**4.19 ± 0.35**	**29.34 ± 0.80**	**23.93 ± 4.03**	**7.27 ± 2.64**	**10.07 ± 3.87**
Epididymis	13.84 ± 0.59	12.09 ± 1.13	14.34 ± 1.45	15.25 ± 1.24	22.44 ± 1.05	12.53 ± 0.87	9.07 ± 1.25
Heart	4.44 ± 0.11	4.06 ± 0.34	4.81 ± 0.21	5.15 ± 0.37	4.37 ± 0.16	3.81 ± 0.15	3.30 ± 0.28
Lung	4.04 ± 0.09	3.79 ± 0.32	4.27 ± 0.18	4.69 ± 0.32	3.95 ± 0.15	3.57 ± 0.09	3.35 ± 0.49
Thymus	4.22 ± 0.14	3.44 ± 0.44	3.99 ± 0.34	4.73 ± 0.49	3.06 ± 0.41	3.44 ± 0.20	2.61 ± 0.35
Liver	5.89 ± 0.20	5.20 ± 0.42	4.85 ± 0.23	6.18 ± 0.35	5.66 ± 0.25	5.18 ± 0.16	3.71 ± 0.14
Pancreas	29.82 ± 1.58	31.56 ± 3.42	41.11 ± 1.50	38.84 ± 3.20	32.86 ± 3.43	36.52 ± 0.88	29.33 ± 1.14
Spleen	4.28 ± 0.23	4.24 ± 0.41	4.84 ± 0.21	5.03 ± 0.35	4.76 ± 0.38	4.04 ± 0.32	3.15 ± 0.20
Kidney	8.64 ± 0.43	8.62 ± 0.64	8.80 ± 0.34	9.84 ± 0.71	9.74 ± 0.44	10.93 ± 0.19	7.35 ± 0.37
Adrenal gland	2.37 ± 0.47	2.59 ± 0.56	5.16 ± 0.79	4.01 ± 0.13	3.36 ± 0.20	2.31 ± 0.39	3.32 ± 0.29
Intestine	5.87 ± 0.24	5.22 ± 0.47	6.20 ± 0.75	4.89 ± 0.41	5.55 ± 0.36	5.83 ± 0.27	4.18 ± 0.21
Muscle	3.81 ± 0.21	4.02 ± 0.61	3.93 ± 0.14	4.28 ± 0.59	3.71 ± 0.13	3.03 ± 0.18	2.86 ± 0.26
Testis	2.76 ± 0.18	2.48 ± 0.24	2.86 ± 0.17	3.25 ± 0.12	3.46 ± 0.07	4.95 ± 0.13	2.86 ± 0.26
Tumor	–	3.56 ± 1.36	6.38 ± 0.52	7.37 ± 0.43	8.55 ± 0.56	8.54 ± 0.05	4.79 ± 0.36
Brain	2.25 ± 0.04	2.20 ± 0.11	2.46 ± 0.46	2.31 ± 0.05	2.44 ± 0.11	2.18 ± 0.06	2.33 ± 0.04

Xie L, *et al. J Immunother Cancer* 2021; 9:e002616. doi: 10.1136/jitc-2021-002616

Supplementary Table 4. Overview of radiotracers for PET imaging of immune cells and the cancer immune response[1-17]. To gauge the tumor elimination efficacy of an immunotherapy, an imaging toolbox has been developed.[3-11 13 14 16-18] However, these imaging tools cannot specifically address the immunoediting response against tumor cells because their target molecules, such as CD3, CD4, CD8, and CD20,[11 19-21] are expressed by both resting and active immune cells, including anti-inflammatory immune cells[8]. Molecules expressed by activated immune cells have also been pursued as imaging targets, for instance, OX40 (CD134), inducible T-cell costimulatory (ICOS), IFN-γ and granzyme B.[10 15 16 18] However, these targets are usually transiently expressed, diffusible, and located primarily in the extracellular matrix. Moreover, it is also worth noting that all the abovementioned methods narrowly focus on tumor sites, which are still too fragmented to produce an understanding of the immunoediting process in a living body. Continuous crosstalk is known to occur between tumors and the host immune system, which shapes antitumor immune responses and determines the efficacy of immunotherapy; hence, methods for imaging off-tumor biomarkers on a whole-body scale could provide a more complete picture of the cancer-immune interaction occurring during immunotherapeutic intervention.

Tracer	Tracer type	Target	Target type	Study model	Therapy involved	Year	Ref.
[18F]FHBG	small molecule	Herpes virus thymidine kinase (sr39TK)	transduced hematopoietic cells	Moloney murine sarcoma	Dexamethasone	2005	1
				human grade IV glioblastoma multiforme	-	2009	2
[18F]FLT	small molecule	thymidine kinase 1 (TK)	lymphocytes	human melanoma	dendritic cell (DC) vaccine	2011	3
89Zr-DFO-CD3	monoclonal antibody	CD3	T lymphocytes	CT26 murine colon carcinoma	anti-CTLA-4	2016	4
89Zr-malDFO-169 cDb	diabody	CD8	CD8+ T cells	EL4/EL4-Ova tumor model and CT26 tumors	antigen-specific adoptive T cell transfer, agonistic antibody therapy (anti-CD137/4-1BB), and checkpoint blockade antibody therapy (anti–PD-L1)	2016	5
[18F]-FAC; [18F]-CFA	small molecule	deoxycytidine kinase (dCK)	CD8+ T lymphocytes	murine glioma cell line GL261; patients with GBM	DC vaccination and/or anti-PD-1 monoclonal antibody (mAb) blockade	2017	6
[18F]FB-IL-2	protein	interleukin-2 receptors (IL-2Rs)	activated CD25C+ T cells	TC-1 tumors	14 Gy local tumor irradiation followed by immunization with 5 ´ 106 SFVeE6,7 particles.	2017	7
89Zr-PEG20-VHH X118	antibody fragment	CD8	CD8+ T cells	B16 melanoma; mesenchymal PB3 cells; epithelial PB2 cells	anti-CTLA4	2017	8
[18F]F-AraG	small molecule	deoxyguanosine kinase (dGK)	activated T cells	mouse model of acute GVHD	allogeneic hematopoietic cell transplantation (HCT)	2017	9
89Zr-anti-IFNg	monoclonal antibody	IFNg	type 1 T helper (Th1)–skewed CD4+ T cells, cytotoxic CD8+ T cells (CTLs), natural killer (NK) cells and NKT cells	TUBO mammary tumors	CpG-ODN; HER2/neu DNA vaccination	2018	10
64Cu-169cDb	diabody	CD8	CD8+ T cells	NDL tumor-bearing FVB mice	CpG + anti-PD-1 antibody administered multiple times or as a single injection in combination or separately	2018	11

89Zr-DFO-CD4 and 89Zr-DFO-CD8a	antibody	CD4 and CD8	CD4+ and CD8a+ tumor-infiltrating lymphocytes (TILs)	B16F10, P815, CT26, MC38, Renca, 4T1, and Sa1N tumors	anti-PD-1 antibody Sym021	2019	12
[64Cu]NOTA-CD8a	antibody	CD8a	CD8a+ T cells	CT26 tumors	external radiation therapy (XRT) in combination with anti-CTLA-4 therapy	2020	13
68Ga-NOTA-GZP	peptide	granzyme B, the serine protease downstream effector of cytotoxic T cells	CD8+ T cells and NK cells	CT26 tumors	anti–PD-1 and anti–CTLA-4 antibody combination treatment	2017	14
				CT26 and MC38 tumors	anti-PD-1 antibody; anti-PD-1 plus anti-CTLA-4 antibodies; anti-PD-1 plus anti-TIM-3 antibodies	2019	15
				CT26 and MC38 tumors	anti-PD-1 plus anti-CTLA-4 antibodies	2020	16
89Zr-DFO-ICOS	monoclonal antibody	inducible T-cell costimulator (ICOS, CD278)	activated cytotoxic T cells, memory T cells, and regulatory T cells	Lewis lung cancer	anti-PD-1 antibody or a STING agonist alone or in combination	2020	17

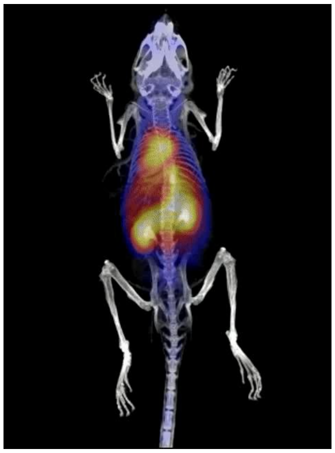

Supplementary Movie 1. **Multidimensional overlaid movie of ^{11}C-L-1MTrp PET imaging following IDO1 blockade-containing combinatorial immunotherapy in mice.** Representative movie from a mouse treated with L-1MTrp + CPA for 0 to 75 min postinjection.

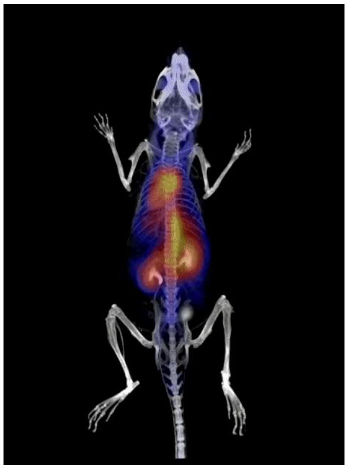

Supplementary Movie 2. **Multidimensional overlaid movie of ^{11}C-L-1MTrp PET imaging following IDO1 blockade-containing combinatorial immunotherapy.** Representative movie from a mouse treated with L-1MTrp + CPA and preinjected with the "cold" IDO1 inhibitor INCB024360 (INCB) for 0 to 75 min postinjection.

Supplementary References

1. Shu CJ, Guo S, Kim YJ, et al. Visualization of a primary anti-tumor immune response by positron emission tomography. *Proc Natl Acad Sci U S A* 2005;102:17412-17.

2. Yaghoubi SS, Jensen MC, Satyamurthy N, et al. Noninvasive detection of therapeutic cytolytic T cells with 18F-FHBG PET in a patient with glioma. *Nat Clin Pract Oncol* 2009;6:53-8.

3. Aarntzen EH, Srinivas M, De Wilt JH, et al. Early identification of antigen-specific immune responses in vivo by [18F]-labeled 3'-fluoro-3'-deoxy-thymidine ([18F]FLT) PET imaging. *Proc Natl Acad Sci U S A* 2011;108:18396-9.

4. Larimer BM, Wehrenberg-Klee E, Caraballo A, et al. Quantitative CD3 PET Imaging Predicts Tumor Growth Response to Anti-CTLA-4 Therapy. *J Nucl Med* 2016;57:1607-11.

5. Tavare R, Escuin-Ordinas H, Mok S, et al. An Effective Immuno-PET Imaging Method to Monitor CD8-Dependent Responses to Immunotherapy. *Cancer Res* 2016;76:73-82.

6. Antonios JP, Soto H, Everson RG, et al. Detection of immune responses after immunotherapy in glioblastoma using PET and MRI. *Proc Natl Acad Sci U S A* 2017;114:10220-25.

7. Hartimath SV, Draghiciu O, van de Wall S, et al. Noninvasive monitoring of cancer therapy induced activated T cells using [(18)F]FB-IL-2 PET imaging. *Oncoimmunology* 2017;6:e1248014.

8. Rashidian M, Ingram JR, Dougan M, et al. Predicting the response to CTLA-4

blockade by longitudinal noninvasive monitoring of CD8 T cells. *J Exp Med* 2017;214:2243-55.

9. Ronald JA, Kim BS, Gowrishankar G, et al. A PET Imaging Strategy to Visualize Activated T Cells in Acute Graft-versus-Host Disease Elicited by Allogenic Hematopoietic Cell Transplant. *Cancer Res* 2017;77:2893-902.

10. Gibson HM, McKnight BN, Malysa A, et al. IFNgamma PET Imaging as a Predictive Tool for Monitoring Response to Tumor Immunotherapy. *Cancer Res* 2018;78:5706-17.

11. Seo JW, Tavare R, Mahakian LM, et al. CD8(+) T-Cell Density Imaging with (64)Cu-Labeled Cys-Diabody Informs Immunotherapy Protocols. *Clin Cancer Res* 2018;24:4976-87.

12. Kristensen LK, Frohlich C, Christensen C, et al. CD4(+) and CD8a(+) PET imaging predicts response to novel PD-1 checkpoint inhibitor: studies of Sym021 in syngeneic mouse cancer models. *Theranostics* 2019;9:8221-38.

13. Kristensen LK, Christensen C, Alfsen MZ, et al. Monitoring CD8a(+) T Cell Responses to Radiotherapy and CTLA-4 Blockade Using [(64)Cu]NOTA-CD8a PET Imaging. *Mol Imaging Biol* 2020;22:1021-30.

14. Larimer BM, Wehrenberg-Klee E, Dubois F, et al. Granzyme B PET Imaging as a Predictive Biomarker of Immunotherapy Response. *Cancer Res* 2017;77:2318-27.

15. Larimer BM, Bloch E, Nesti S, et al. The Effectiveness of Checkpoint Inhibitor Combinations and Administration Timing Can Be Measured by Granzyme B PET Imaging. *Clin Cancer Res* 2019;25:1196-205.

16. LaSalle T, Austin EE, Rigney G, et al. Granzyme B PET imaging of immune-mediated tumor killing as a tool for understanding immunotherapy response. *J Immunother Cancer* 2020;8

17. Xiao Z, Mayer AT, Nobashi TW, et al. ICOS Is an Indicator of T-cell-Mediated Response to Cancer Immunotherapy. *Cancer Res* 2020;80:3023-32.

18. Alam IS, Mayer AT, Sagiv-Barfi I, et al. Imaging activated T cells predicts response to cancer vaccines. *J Clin Invest* 2018;128:2569-80.

19. Beckford Vera DR, Smith CC, Bixby LM, et al. Immuno-PET imaging of tumor-infiltrating lymphocytes using zirconium-89 radiolabeled anti-CD3 antibody in immune-competent mice bearing syngeneic tumors. *PloS one* 2018;13:e0193832.

20. Freise AC, Zettlitz KA, Salazar FB, et al. ImmunoPET Imaging of Murine CD4(+) T Cells Using Anti-CD4 Cys-Diabody: Effects of Protein Dose on T Cell Function and Imaging. *Mol Imaging Biol* 2017;19:599-609.

21. James ML, Hoehne A, Mayer AT, et al. Imaging B Cells in a Mouse Model of Multiple Sclerosis Using 64Cu-Rituximab PET. *J Nucl Med* 2017;58:1845-51.
