# Supplementary figures and images for: Off-tumor IDO1 target engagements determine the cancer-immune set point and predict the immunotherapeutic efficacy
Source: J Immunother Cancer. 2021 Jun 20;9(6):e002616. doi: 10.1136/jitc-2021-002616 (PMC8237741; doi:10.1136/jitc-2021-002616)

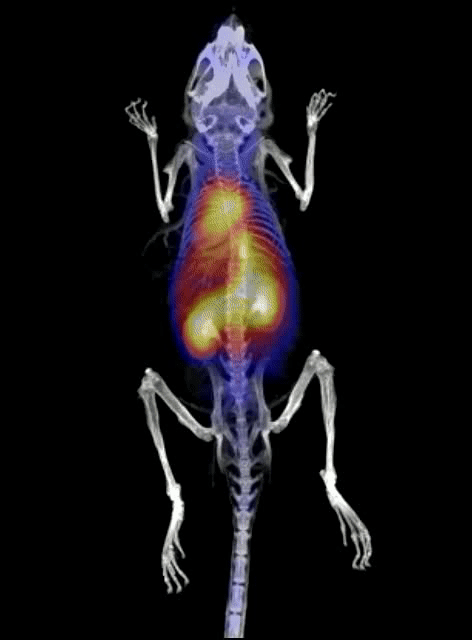

Supplement: Supplementary data [file jitc-2021-002616supp002.gif]

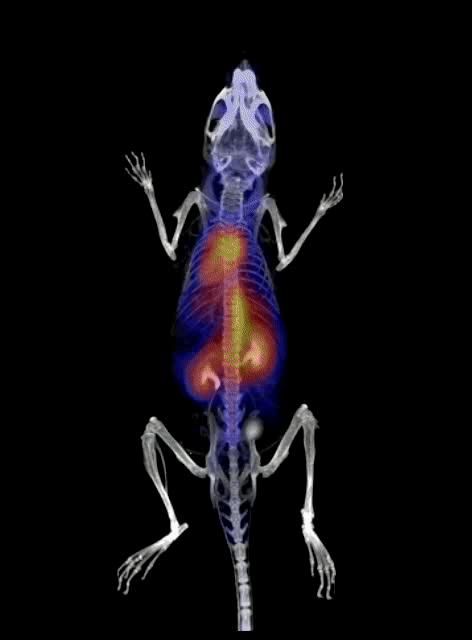

Supplement: Supplementary data [file jitc-2021-002616supp003.gif]
